# A fast Fourier convolutional deep neural network for accurate and explainable discrimination of wheat yellow rust and nitrogen deficiency from Sentinel-2 time series data

**DOI:** 10.3389/fpls.2023.1250844

**Published:** 2023-10-04

**Authors:** Yue Shi, Liangxiu Han, Pablo González-Moreno, Darren Dancey, Wenjiang Huang, Zhiqiang Zhang, Yuanyuan Liu, Mengning Huang, Hong Miao, Min Dai

**Affiliations:** ^1^ School of Electronic and Electrical Engineering, University of Leeds, Leeds, United Kingdom; ^2^ Department of Computing and Mathematics, Manchester Metropolitan University, Manchester, United Kingdom; ^3^ School of Biology, University of Cordoba, Cordoba, Spain; ^4^ Aerospace Information research Institute, Chinese Academy of Sciences (CAS), Beijing, China; ^5^ Department of Computer Science, The University of Manchester, Manchester, United Kingdom; ^6^ School of Computing, Beijing University of Technology, Beijing, China; ^7^ School of Mechanical Engineering, Yangzhou University, Yangzhou, China

**Keywords:** deep learning, time-series analysis, precision agriculture, Sentinel-2, plant protection, winter wheat

## Abstract

**Introduction:**

Accurate and timely detection of plant stress is essential for yield protection, allowing better-targeted intervention strategies. Recent advances in remote sensing and deep learning have shown great potential for rapid non-invasive detection of plant stress in a fully automated and reproducible manner. However, the existing models always face several challenges: 1) computational inefficiency and the misclassifications between the different stresses with similar symptoms; and 2) the poor interpretability of the host-stress interaction.

**Methods:**

In this work, we propose a novel fast Fourier Convolutional Neural Network (FFDNN) for accurate and explainable detection of two plant stresses with similar symptoms (i.e. Wheat Yellow Rust And Nitrogen Deficiency). Specifically, unlike the existing CNN models, the main components of the proposed model include: 1) a fast Fourier convolutional block, a newly fast Fourier transformation kernel as the basic perception unit, to substitute the traditional convolutional kernel to capture both local and global responses to plant stress in various time-scale and improve computing efficiency with reduced learning parameters in Fourier domain; 2) Capsule Feature Encoder to encapsulate the extracted features into a series of vector features to represent part-to-whole relationship with the hierarchical structure of the host-stress interactions of the specific stress. In addition, in order to alleviate over-fitting, a photochemical vegetation indices-based filter is placed as pre-processing operator to remove the non-photochemical noises from the input Sentinel-2 time series.

**Results and discussion:**

The proposed model has been evaluated with ground truth data under both controlled and natural conditions. The results demonstrate that the high-level vector features interpret the influence of the host-stress interaction/response and the proposed model achieves competitive advantages in the detection and discrimination of yellow rust and nitrogen deficiency on Sentinel-2 time series in terms of classification accuracy, robustness, and generalization.

## 1 Introduction

The plant stress caused by unfavorable environmental conditions (e.g., a lack of nutrients, insufficient water, disease, or insect damage), if left untreated, will lead to irreversible damage and decreases in plant production. Early accurate detection of plant stress is essential to be able to respond with appropriate interventions to reverse stress and minimize yield loss. Recent advances in remote sensing with enhanced spatial, temporal, and spectral capacities, combined with deep learning, have offered unprecedented possibilities for rapid noninvasive stress detection in a fully automated and reproducible manner (Ji et al., 2018; Wang et al., 2020). Currently, the deep learning models have been proven effective in remote sensing time series analysis of plant stresses (Golhani et al., 2018; Abdur Rehman et al., 2019). One-dimensional convolutional neural network (1D-CNN) and 2D-CNN with convolutions were applied either in the spectral domain or in the spatial domain (Kussul et al., 2017; Scarpa et al., 2018). In addition, 3D-CNNs were also used across spectral and spatial dimensions (Li et al., 2017; Hamida et al., 2018). These models do not consider temporal information. Meanwhile, temporal 1D-CNNs were proposed to handle the temporal dimension for general time series classification (Wang et al., 2017) and recurrent neural network (RNN)-based models to extract features from multi-temporal observation by leveraging the sequential properties of multispectral data and combination of RNN (Kamilaris and Prenafeta-Boldú, 2018) and 2D-CNNs where convolutions were applied in both temporal and spatial dimensions (Zhong et al., 2019). These preliminary works highlight the importance of temporal information that can improve the classification accuracy performance. Although the existing works are encouraging, they suffer several limitations: 1) over-fitting and uncertainty caused by noisy data involved in the remote sensing time series; 2) computing inefficiency and inaccuracy caused by the convolutional operations that are applied to all layers, particularly with the increase of size of images and the kernel. In particular, for the classification of multi-plant stresses, similar symptoms always lead to confusion during classification, as most of the local features are extracted from the neighbor time steps. Therefore, a more effective denoise operator and larger receptive fields for the extraction of the global biological responses at various timescales are highly desired.

One solution is to prefilter the photochemical information from satellite time series and change the domain through Fourier transform to model the part-to-whole relationship between the photochemical features and specific plant stress in the frequency domain. This is because the convolution operation in the spatial domain is the same as the point-by-point multiplication in the Fourier domain. According to the Fourier theory, Fourier transform provides an effective perception operation with a nonlocal receptive field. Unlike existing CNNs where a large-sized kernel is used to extract local features, Fourier transforms with a small-sized kernel can capture global information. Therefore, the Fourier kernel has great potential in replacing the traditional convolutional kernel in remote sensing time series analysis without any additional effort (Yi et al., 2023). For example, Chen et al. (2023) designed a Fourier domain structural relationship analysis framework to exploit both modality-independent local and nonlocal structural relationships for unsupervised change detection. However, the existing Fourier operators can only be sparsely inserted into the deep learning network pipeline due to their expensive computational cost. Therefore, the fast Fourier transform (FFT) is an effective way to extract the global feature responses from satellite image time series (Nguyen et al., 2020). For example, Awujoola et al. (2022) proposed a multi-stream fast Fourier convolutional neural network (MS-FFCNN) by utilizing the FFT instead of the traditional convolution; it lowers the computing cost of image convolution in CNNs, which lowers the overall computational cost. Lingyun et al. (2022) designed a spectral deep network combining fast Fourier convolution (FFC) and classifier by extending the receptive field. Their results demonstrated that the features around the object provide the explainable information for small object detection.

Although the effectiveness of Fourier-based convolution has been proven by many studies, few studies have done in the multiple plant stress detection from remote sensing data. In this work, we have proposed a novel fast Fourier convolutional deep neural network (FFCDNN) for accurate and early efficient detection of plant stress with an initial focus on wheat yellow rust (*Puccinia striiformis*) and nitrogen deficiency. The proposed model significantly reduces the computing cost with improved accuracy and interpretability. Specifically, a new FFT kernel is proposed as the basic perception unit of the network to extract the stress-associated biological dynamics with various timescales; and then the extracted biological dynamics are encapsulated into a series of high-level featured vectors representing the host–stress interactions specific to different stresses; finally, a nonlinear activation function is designed to achieve the final decision of the classification. The proposed model has been evaluated with ground truth data under both the controlled and natural conditions.

The rest of this work is organized as follows. *The Related Work* section introduces related works on existing methods of multiple plant disease classification. *The Proposed Fast Fourier Convolutional Deep Neural Network* section details the proposed approach. The *Materials and Experiments* section presents the material and experiment details. The *Results and Discussion* section illustrates the experimental evaluation results. Finally, the *Conclusion* section concludes the work.

## 2 The related work

### 2.1 Plant photochemical information filter from satellite images

The newly launched satellite sensors (e.g., Sentinel-2 and WorldView-3) provide the promising Earth observation (EO) dataset for improved plant photochemical estimation (Xie et al., 2018) wherein leaf chlorophyll content (LCC), canopy chlorophyll content (CCC), and leaf area index (LAI) are the most popular remotely retrievable indicators for detecting and discriminating plant stresses (Haboudane et al., 2004; Elarab et al., 2015). Among these indicators, the LCC time series is a key biochemical dynamics for the stress-associated foliar component changes without (or partly) the effects from soil background and canopy structure. Estimating LCC requires remote sensing indicators that are sensitive to the LCC but, at the same time, are insensitive to LAI and background effects (Elarab et al., 2015). On the other hand, the LAI is one of the critical biophysics-specific proxies used in characterizing the canopy architecture variations that respond to the apparent symptom caused by specific stress (Li et al., 2018). By contrast, CCC is determined by the LAI and LCC, expressed per unit leaf area, which retains multicollinearity with them and hard to be used in separating the stress-induced biochemical changes from the biophysical impacts. Therefore, the LCC and LAI are regarded as a pair of independent variables for filtering the biochemical information between the different plant stresses (Zhang et al., 2012; Shi et al., 2017a).

Regarding the filter methods, by using the reflectance in red-edge regions, there are two methods used in LAI and LCC estimation for minimizing the saturation effect and soil background-associated noises: 1) the vegetation index method (Haboudane et al., 2002; Li et al., 2018); 2) the radiative transfer models (RTMs) (Darvishzadeh et al., ????; Sehgal et al., 2016). For example, Clevers and Gitelson (2013) tested and compared the performance of the red-edge chlorophyll index (CIred-edge) and green chlorophyll index (CIgreen) on the Sentinel-2 bands, and their results indicated that the setting of Sentinel-2 bands is well positioned for deriving these indices on LCC estimation. Punalekar et al. (2018) developed a PROSAIL-based model to estimate LAI and biomass on the Sentinel-2 bands, and the yielded LAI values are in agreement with the ground truth LAI measurements. However, the simple use of the remotely estimated LAI and LCC cannot easily represent the nonlinear host–stress interactions of plant stresses.

### 2.2 Plant stress detection methods

Currently, there are two types of methods widely used in extracting the interpretable agent features for plant stresses from satellite imagery, including the biological methods and the deep learning-based methods.

#### 2.2.1 Biological methods

Studies have shown that biological models can be used to map within-field crop stress variability (Ryu et al., 2020; Zhou et al., 2021a). This is possible because the infestation of crop stresses often leads plants to close their stomata, decreasing canopy stomatal conductance and transpiration, which in turn raises foliar biophysical and biochemical variations (Tan et al., 2019). However, plant stress involves complicated biophysical and biochemical responses, which demands the stress-specific biological index. For instance, LAI is a direct indicator of plant canopy structure features (Ihuoma and Madramootoo, 2019). Stressed plants will lead to fluctuations on plant LAI time series with different patterns, which will raise the higher radiations of a stressed crop (Ballester et al., 2019). Jiang et al. (2020) proposed two LAI-derived soil water stress functions in order to quantify the effect of soil water stress on the processes of leaf expansion and leaf senescence caused by the stresses. Their results showed that the LAI-based model is sensitive to the stress-derived leaf expansion. Zhu et al. (2021) developed a vegetation index-derived model from the observed hyperspectral data of winter wheat to detect plant salinity, and the results show that the salt-sensitive blue, red-edge, and near-infrared wavebands have great performances on the detection of plant salinity stress.

Unlike the LAI, the photochemical associated indices directly account for leaf physiological changes such as photosynthetic pigment changes (Gerhards et al., 2019). Photochemical reflectance is the dominant factor determining leaf reflectance in the visible wavelength (400 nm–700 nm), with chlorophyll considered the most relevant photochemical index for crop stress diagnosis (Zhou et al., 2021b). Under prolonged infestations, LCC often decreases, leading to a reduction in green reflection and an increase in blue and red reflections. The spectral radiation characteristics between the red and near-infrared regions are sensitive to LCC and CCC. The ratio of red and near-infrared has shown a strong sensitivity to the crop stress-associated chlorophyll content changes (Ryu et al., 2020). Cao et al. (2019) compared the feasibility of the LCC, net photosynthesis rate, and maximum efficiency of the photosystem on the detection of crop heat stress, and their findings suggest that the maximum efficiency of the photosystem was the most sensitive remote sensing agent to heat stress and had the ability to indicate the start and end of the stress at the slight level or the early stage. Shivers et al. (2019) used the visible-shortwave infrared (VSWIR) spectra to model the non-photosynthetic vegetation and soil background from the airborne visible/infrared imaging spectrometer (AVIRIS), and their findings revealed that the increase in temperature residuals is highly consistent with the infestation of crop stresses.

#### 2.2.2 Machine/deep learning-based methods

Although many studies have been focusing on crop stress detection using biological characteristics, most of the applications require self-adjusted algorithms to improve the robustness and generalization of the model for complicated nature conditions. Among the crop stress detection techniques, machine learning and deep learning have played a key role. For machine learning approaches, supervised models have been proven effective in data mining from the training dataset (Kaneda et al., 2017). The data flow in the machine learning models includes feature extraction, data assimilation, optimal decision boundary searching, and classifiers for stress diagnosis, whereas supervised learning deals with classification issues by representing the labeled samples. Such models aim to find the optimal model parameters to predict the unlabeled samples (Harrington, 2012).

Deep learning has many neural layers that transform the sensitive information from input to output (i.e., healthy or stressed). The most applied perception neural unit is the convolutional neural unit in crop stress detection (Fuentes et al., 2017; Krishnaswamy Rangarajan and Purushothaman, 2020). Generally, the convolutional neural unit consists of dozens of layers that process the input information with convolution kernel. In the area of crop stress detection, deep learning contributed significantly to the analysis of plant stress high-level features (Jin et al., 2018). In crop stress image classification, the multisource images are usually used as input to extract the stress dynamics during their development, and a diagnostic decision is used as output (e.g., healthy or diseased) (An et al., 2019; Cruz et al., 2019). Barbedo (2019) developed a convolutional deep learning model to classify individual lesions and spots on plant leaves. This model has been successfully used in the identification of multiple diseases; the accuracy obtained in this model was 12% higher than that of traditional models. Lin et al. (2019) applied a convolutional kernel-based U-Net to segment powdery mildew-infected cucumber leaves. The proposed binary cross-entropy loss function is used to magnify the loss of the powdery mildew-stressed pixels, and the average accuracy for the powdery mildew detection reaches 96.08%.

### 2.3 Interpretability of deep learning-based models

Although the deep learning models have been successfully applied for vegetation stress-monitoring applications, most of the existing deep learning-based approaches have difficulty in explaining plant biophysical and biochemical characteristics due to their black box representations of the features extracted from intermediate layers (Shi et al., 2021). Thus, the interpretability of deep models has become one of the most active research topics in the remote sensing-based crop stress diagnosis, which can enhance and improve the robustness and accuracy of models in the vegetation-monitoring applications from the biological perspective of target entities (Brahimi et al., 2019; Too et al., 2019).

Recently, the model interpretability used to disclose the intrinsic learning logic for detection and discrimination of plant stresses has received growing attention (Lillesand et al., 2015). In other words, the interpretability that illustrates the performance of the model on characterizing the specific host–stress interaction guarantees the generalization ability of the model for practice usages. Among the existing models, visualization of the feature representation is the most direct method for improving model interpretability. For example, Behmann et al. (2014) proposed an unsupervised model for early detection of the drought stress in barley wherein the intermediate features produced by this model highly related with the sensitive spectral bands for drought stress. Another way to improve the interpretability of deep learning models is to construct the network architecture that can bring the network an explicit semantic meaning. For example, Shi et al. (2021) developed a biologically interpretable two-stage deep neural network (BIT-DNN) for the detection and classification of yellow rust from the hyperspectral imagery. Their findings demonstrate that the BIT-DNN showed great advantages in terms of accuracy and interpretability.

### 2.4 Fast Fourier transform

Traditional receptive fields act only on the central region to extract localized features related to the target of interest. This limits the necessity of large convolutional kernel on global feature extraction. Recently, there is an increasing interest in applying Fourier transform to deep neural networks to capture global features. As mentioned in the *Introduction* section, Fourier transform provides an effective perception operation with nonlocal receptive fields. Unlike existing CNNs where a large-sized kernel is used to extract local features, Fourier transform with a small-sized kernel is able to capture global information. For example, Rippel et al. (2015) proposed a Fourier transformation pooling layer that performs like principle component extraction by constructing the representation in the frequency domain. Chi et al. (2019) proposed to integrate the Fourier transforms into a series of convolution layers in the frequency domain.

FFT-based deep learning models use the time-frequency analysis methods to extract the low-frequency host–stress interaction by limiting the high-frequency noises in the frequency domain space (Jakubauskas et al., 2002; Behmann et al., 2014; Ashourloo et al., 2016; Mahlein et al., 2017). FFT is a useful harmonic analysis tool, which has been widely used in reconstruction of vegetation index time series (Roy and Yan, 2020), curve smoothing (Bradley et al., 2007; Shao et al., 2016), and ecological and phenological applications (Jakubauskas, 2002; Sakamoto et al., 2005; Jong et al., 2011). FFT maps the satellite time series signals into superimposed sequences of cosines waves (terms) with variant frequencies, each component term accounting for a percentage of the total variance in the original time series data (Jakubauskas et al., 2002). This process facilitates the recognition of subtle patterns of interest from the complex background noises, which degrade the spectral information required to capture vegetation properties (Huang et al., 2018; Shanmugapriya et al., 2019). For example, El Jarroudi et al. (2017) used the FFT method to characterize temporal patterns of the fungal disease on winter wheat between the observation sites and then achieved the fungal disease monitoring and forecasting at the regional level. Our work advances the abovementioned research front via designing a novel fast Fourier convolutional operation unit that simultaneously uses spatial and temporal information for achieving global feature extraction during the learning process.

## 3 The proposed fast fourier convolutional deep neural network

To address the challenge of the misclassification of the different plant stresses with similar symptoms, we propose a novel FFC operator to efficiently implement nonlocal receptive fields and fuse the extracted biological information with various timescales in the frequency domain, and then, a new deep learning architecture is developed to retrieve the host–stress interaction and achieve a high-accuracy classification. In this section, we describe the main framework of the proposed FFCDNN in the context of multiple plant stress discrimination from the agent-based biological dynamics.

### 3.1 The network architecture of the proposed FFCDNN

**Figure 1:**
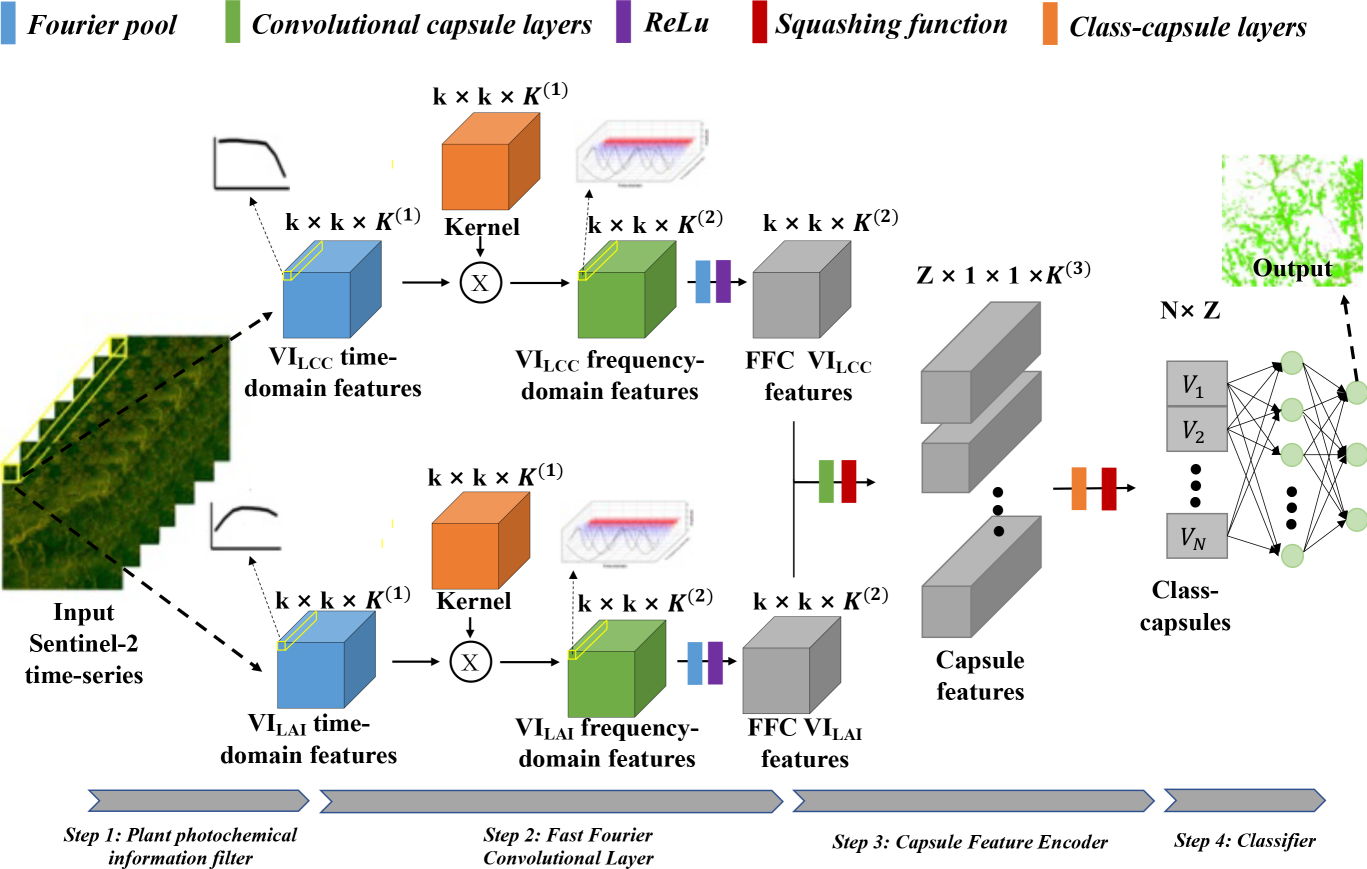
The workflow of the FFCDNN, Fast Fourier Convolutional Deep Neural Network framework for the discrimination of multiple plant stresses from Sentinel-2 time series.

**Figure 1** depicts the main framework of the proposed FFCDNN for multiple crop stress discrimination in the context of Sentinel-2-derived biological agents (i.e., *V I_LAI_* and *V I_LCC_*). To be specific, a branch structure is designed to respectively prefilter the biochemical dynamics represented by *V I_LAI_* and *V I_LCC_* time series. For each of the branches, the Fourier kernel is set as the same size as the input size of *VI_LAI_* and *V I_LCC_* time domain (time series) patches, with a size of *k* × *k* × *K* ^(1)^; then, the Fourier kernel is pont-wised multiplied by the input biological agent patches. After the Fourier convolution is performed, the ReLU function is implemented to calculate the *V I_LAI_* and *V I_LCC_* time series magnitude in the frequency domain containing stress-associated biological responses, and the activation feature map, with a size of *k* × *k* × *K* ^(2)^, is conducted with Fourier pool layer to highlight the most important stress information and downsampling the feature map.

Subsequently, the *V I_LAI_* and *V I_LCC_* feature maps are sent to the hierarchical structure of the class capsule blocks in order to build the part-to-whole relationship and to generate the hierarchical vector features for representing the high-level stress–pathogen interaction. Finally, a decoder is employed to predict the classes based on the length and direction of the hierarchical vector features in the feature space. The detailed information for the model blocks is described below:

#### 3.1.1 Plant photochemical information filter

In this study, an agent-based photochemical information prefilter is set as the preprocessing operator for the input satellite time series. Based on the benchmark study of the existing vegetation agent models for LAI and LCC estimation shown in Appendix A, we use the weighted difference vegetation index (WDVI)-derived LAI, defined as *V I_LAI_*, and transformed chlorophyll absorption in the reflectance index/optimized soil-adjusted vegetation index (TCARI/OSAVI)-derived LCC, defined as *V I_LCC_*, as the optimal plant photochemical information prefilter on Sentinel-2 bands. And then, the *V I_LAI_* and *V I_LCC_* time series will be used as the biological agents of the plant canopy structure and plant biochemical state in the follow analysis.

#### 3.1.2 Fast Fourier convolutional layer

The input biological agent (i.e., *V I_LAI_* or *V I_LCC_*) dynamics extracted from the Sentinel-2 time series can be viewed as sample patch *k* × *k* pixel vectors. Each of the pixels represents a class with *K* ^(1)^ time series channels. Then, the 3D patches with a size of *k* × *k* × *K* ^(1)^ are extracted as the input of the past Fourier convolution layer.

The FFC is used to decompose the biological agent time series into a series of frequency components with various timescales based on the FFT. Mathematically, FFT decomposes the original time series signal *f*(*t*) to the frequency domain by the linear combination of trigonometric functions as follows:


(1) $$F\left(\varpi \right)=\int_{-\infty }^{+\infty }f\left(t\right)e^{-i\varpi t}dt$$

where ϖ is the frequency, F(ϖ) is the Fourier coefficient with frequency ϖ , and *i* is the unit of the imaginary number. It is customary to use a discrete form as follows:


(2) $$F{\left(x\right)}_{k\times k}=\frac{1}{K^{1}}\sum_{n=0}^{K^{1}-1}{x_{n}e}^{-\frac{2\prod xni}{K^{1}}}$$

where *x* = 0,1,2*,…N* – 1 and *N* is the length of the time series.

Among the frequency-domain components of the biological agents of *V I_LAI_* and *V I_LCC_* dynamics, the low-frequency components always indicate the soil background or phenological characteristics of the ground entities. The high-frequency region generally represents environmental noises, such as land cover variations or illumination inconsistency. Therefore, considering that the infestation and development of yellow rust and nitrogen deficiency are continuous biological processes on the proxies of *V I_LAI_* and *V I_LCC_*, we hypothesize that the medium-frequency region represents the yellow rust- and nitrogen deficiency-associated *V I_LAI_* and *V I_LCC_* fluctuations. Thus, the yellow rust- and nitrogen deficiency-associated responses can be characterized from the background and environmental noises by an optimized activation function. In this study, the ReLU activation function is implemented to calculate the *V I_LAI_* and *V I_LCC_* time series magnitude in the medium-frequency region, and the activation feature map, with a size of *k* × *k* × *K* ^(2)^, is conducted with Fourier pool layer to extract the sensitive *V I_LAI_* and *V I_LCC_* response in the frequency domain and output the FFC features.

#### 3.1.3 Capsule feature encoder

**Figure 2:**
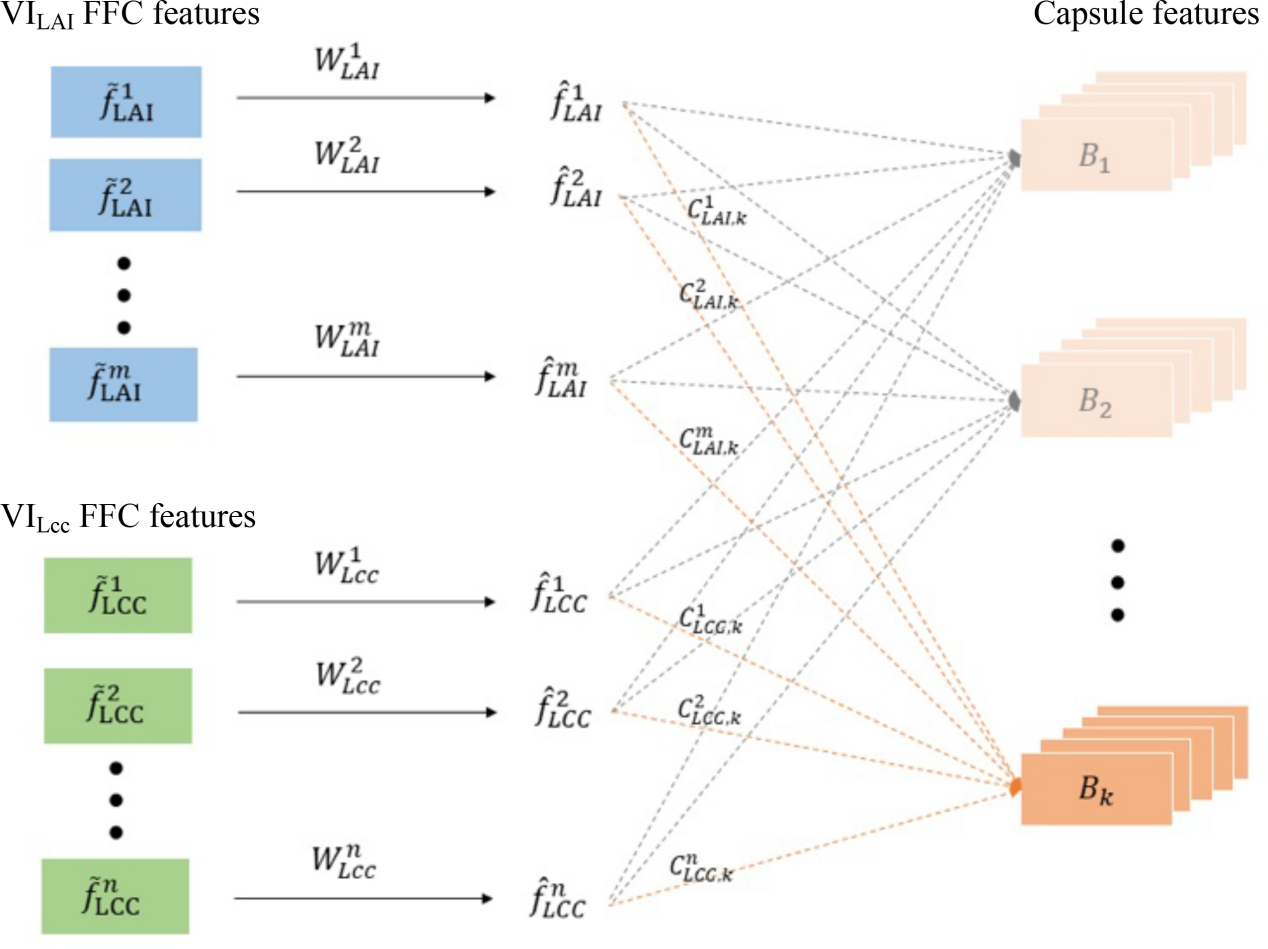
The dynamic routing optimization between the FFC scalar features and the capsule vector features.

Considering the host–stress interaction of the plant stresses is a complex biological process. Therefore, modeling the part-to-whole relationship is the most significant evidence for detection and discrimination of plant stresses. We develop a capsule feature encoder to rearrange the extracted *V I_LAI_* and *V I_LCC_* FFC features, which are the scalar features, into the joint capsule vector features. These joint vector features represent the hierarchical structure of the *V I_LAI_* and *V I_LCC_* responses to the specific plant stress. It is noteworthy that the extracted *V I_LAI_* and *V I_LCC_* scalar FFC features themselves respectively represent the biophysical and biochemical response to the plant stress development. Therefore, the joint vector features have great performance to characterize the intrinsic entanglement of host–stress interactions. In order to optimize the learning process between the FFC scalar features and the capsule vector features, and dynamic routing algorithm is introduced as shown in **Figure 2**.

Specifically, the *V I^LAI^* and *V I^LCC^* FFC features, $\left\{{\bar{f}}_{LAI}^{1},{\bar{f}}_{LAI}^{2},\ldots ,{\bar{f}}_{LAI}^{m},{\bar{f}}_{LCC}^{1},{\bar{f}}_{LCC}^{2},\ldots {\bar{f}}_{LCC}^{n}\right\}$ , are firstly normalized by using the normalization weights $W\in \left\{W_{LAI}^{1},W_{LAI}^{2},\ldots W_{LAI}^{m},W_{LCC}^{1},W_{LCC}^{2},\ldots W_{LCC}^{n}\right\}$ . This step smooths the feature values and makes them obey a normal distribution. In addition, this normalization operation is helpful for retraining the vanishing gradients in the back-propagation progress. After that, the normalized FFC features, $\left\{{\hat{f}}_{LAI}^{1},{\hat{f}}_{LAI}^{2},\ldots ,{\hat{f}}_{LAI}^{m},{\hat{f}}_{LCC}^{1},{\hat{f}}_{LCC}^{2},\ldots ,{\hat{f}}_{LCC}^{n}\right\}$ , are rearranged that into *K* ^3^ capsule features with the coupling coefficients of *c*. Here, *c* is a series of trainable parameters that encodes the part–whole relationships between the FFC scalar features and the capsule vector features. The translation and orientation of the capsule vector feature represent the class-specific hierarchical structure characteristics in terms of *V I_LAI_* and *V I_LCC_* responses in the frequency domain, while its length represents the degree a capsule is corresponding to a class. To measure the length of the output vector as a probability value, a nonlinear squash function is used as follows:


(3) $${\overset{⌣}{u}}_{m}=\frac{{∥u_{m}∥}^{2}}{1+{∥u_{m}∥}^{2}}\cdot \frac{u_{m}}{∥u_{m}∥}$$

where ${\overset{⌣}{u}}_{m}^{\left(l\right)}$ is the scaled vector of $X_{out}^{2}$ . This function compresses the short vector features to zero and enlarges the long vector features to a value close to 1. The final output is denoted as $X_{out}^{3}\in {\mathbb{R}}^{Z\times 1\times 1\times K}$ .

Finally, the *K* ^3^ capsule features will be weightily combined into *Z* class capsules, and the final outputs are the class-wised biologically composed feature = $\left\{V_{1},V_{2},\ldots ,V_{Z}\right\}$ . In this study, *Z* is 3 because of the three interested classes (i.e., healthy wheat, yellow rust, and nitrogen deficiency).

#### 3.1.4 Classifier

Based on the characteristics of the class-capsule feature vectors, a classifier is defined to achieve the final detection and discrimination. This classifier is composed of two layers: an activation layer and a classification layer.

Specifically, the active function is defined as follows:


(4) $${\hat{V}}_{h}=\frac{{∥V_{h}∥}^{2}}{1+∥V_{h}∥}\cdot \frac{V_{h}}{∥V_{h}∥}$$

where *V_h_* is the class-capsule feature corresponding to class h≦Z.║·║ indicates the operator of 1-norm. In fact, the orientation of the ${\hat{V}}_{h}$ represents the instantiation parameters of the biological responses for the class *h*, and the length represents the membership that the feature belongs to class *h*. And then, an argmax function is used to achieve the final classification by seeking the largest length of ${\hat{V}}_{h}$ . The argmax function is defined as follows:


(5) $$\underset{h}{argmax}O_{i,j}^{5}=\left\{h|\forall g:║Vg║<║V_{h}║\right\}$$

## 4 Materials and experiments

In this study, we use nitrogen deficiency and the yellow rust as the study cases for model testing and evaluation. In order to comprehensively test and evaluate the classification accuracy, robustness, and generalization of the proposed model, we collected two types of the data: 1) the high-quality labeled dataset under the controlled field conditions; 2) the ground survey dataset under the natural field conditions. The former is used for training and optimizing the proposed model, and the latter is used for testing and evaluating the generalization and transferability of the well-trained model in the actual application cases. The detailed information is described as follows:

### 4.1 Study sites

To avoid the fungus contamination on the other groups, we respectively carried out two independent experiments under similar environmental conditions by recording continuous *in-situ* observations of: a) yellow rust infestation from 20 April to 25 May 2017 at the Scientific Research and Experimental Station of Chinese Academy of Agricultural Science (39°30′40″N, 116°36′20″E) in Langfang, Hebei province, and b) nitrogen deficiency at the National Experiment Station for Precision Agriculture (40°10′6″N, 116°26′3″E) in Changping District, Beijing, China. The measurement strategies focused on eight key wheat growth stages (i.e., jointing stage, flag leaf stage, heading stage, flowering stage, early grain-filling stage, mid grain-filling stage, late grain-filling stage, and harvest stage). The detailed observation dates and the canopy photographs were listed in **Table 1**. The same experiments were repeated from 18 April to 31 May 2018.

**Table 1:** The state of vegetation at each measurement date.

Location (year)	Type	Day after treatment (DAT)			
Langfang 2017	H	7(Apr.20) 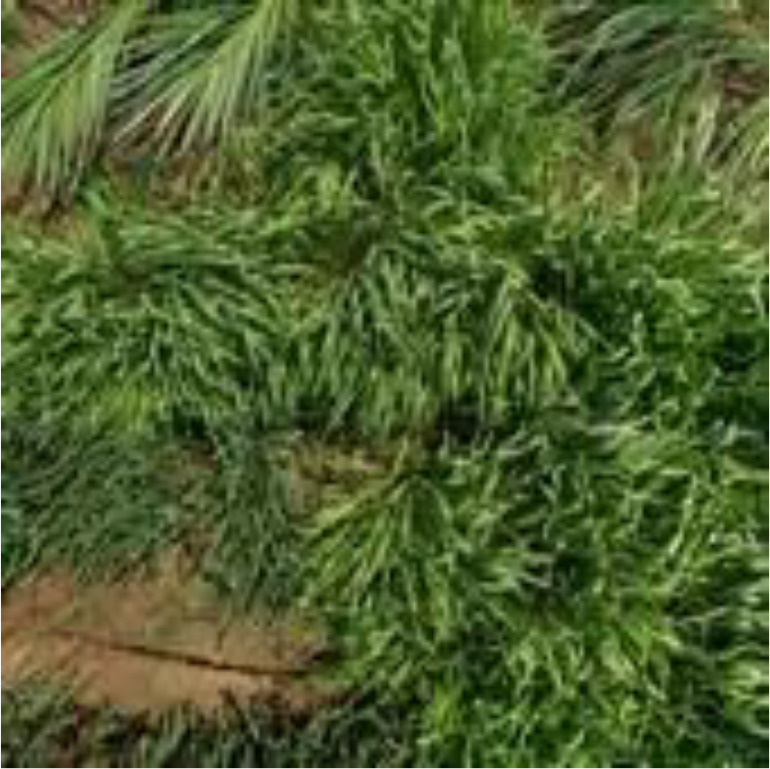	14(Apr.27) 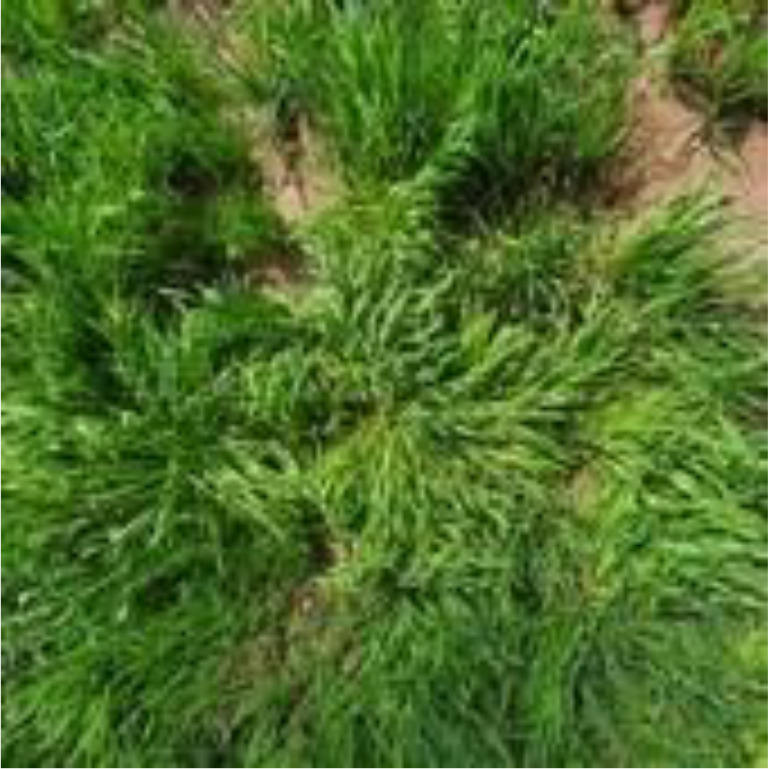	23(May.6) 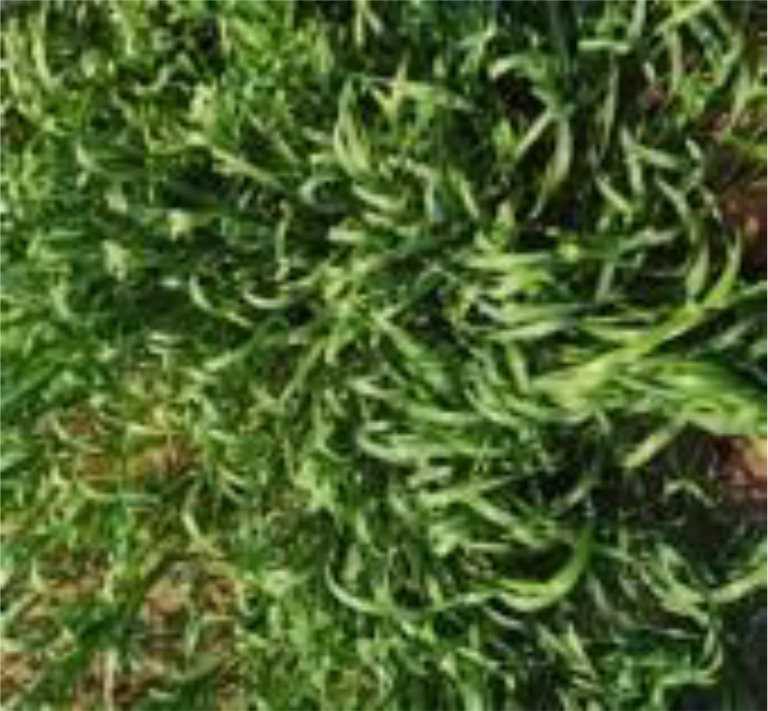	27(May.10) 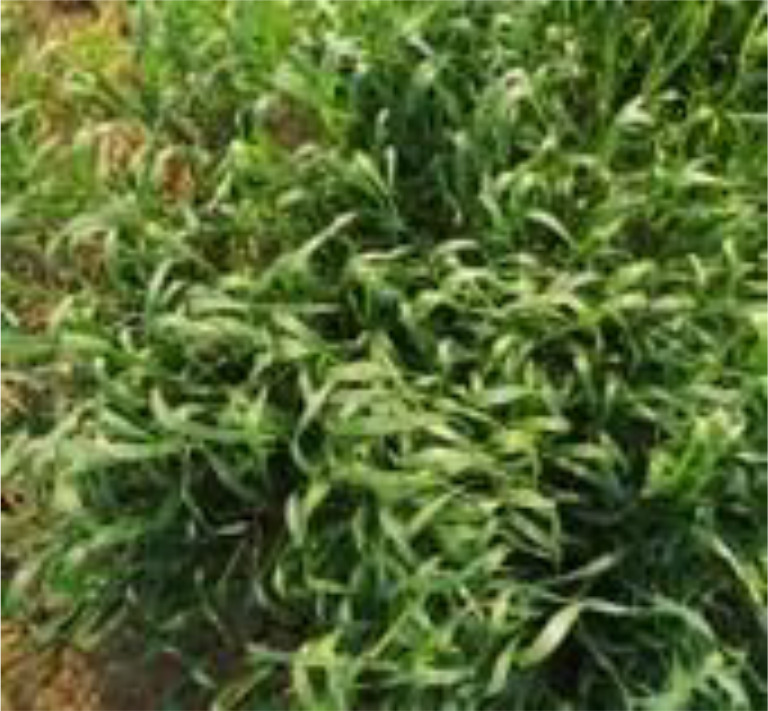
	YR	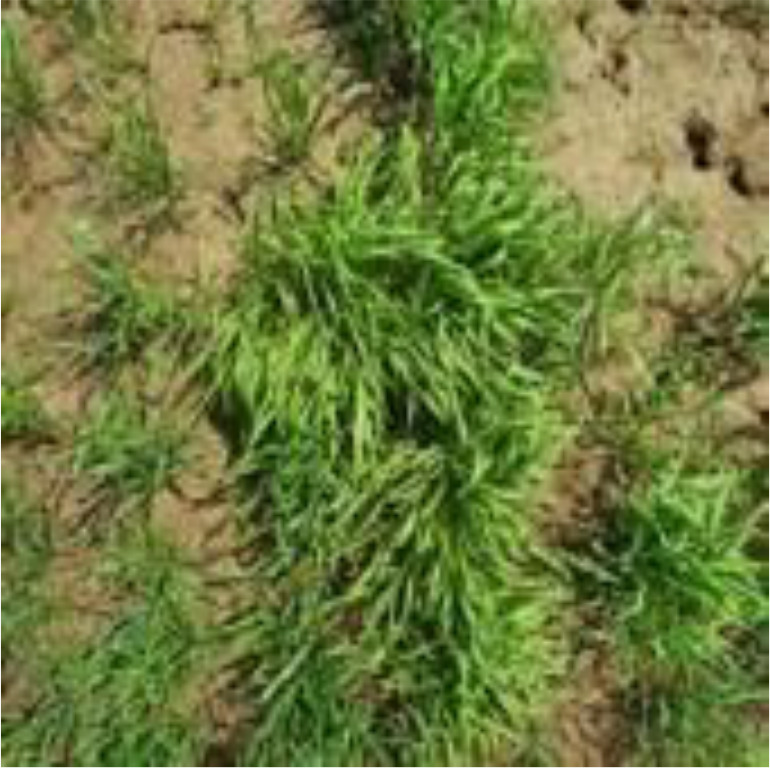	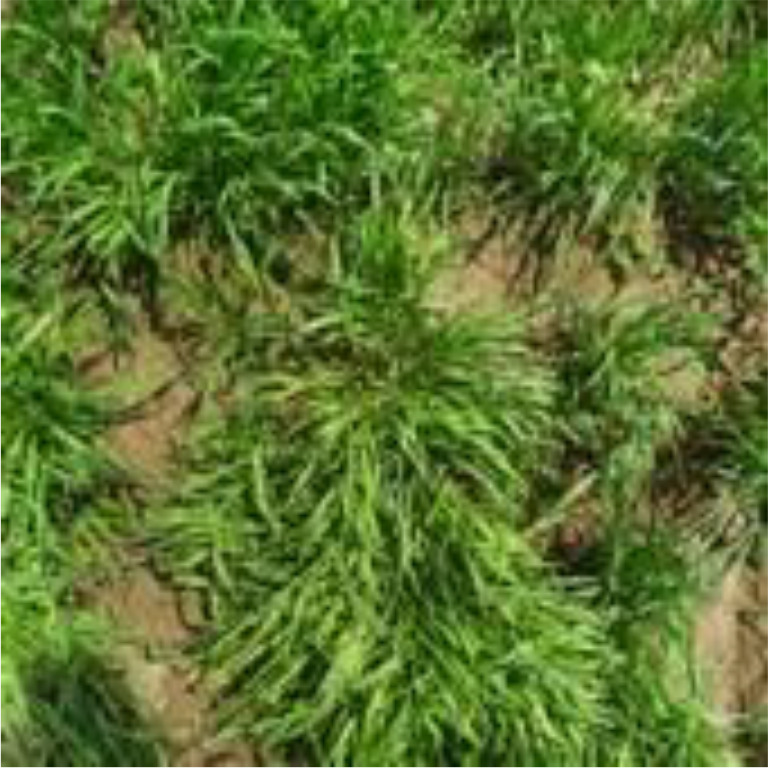	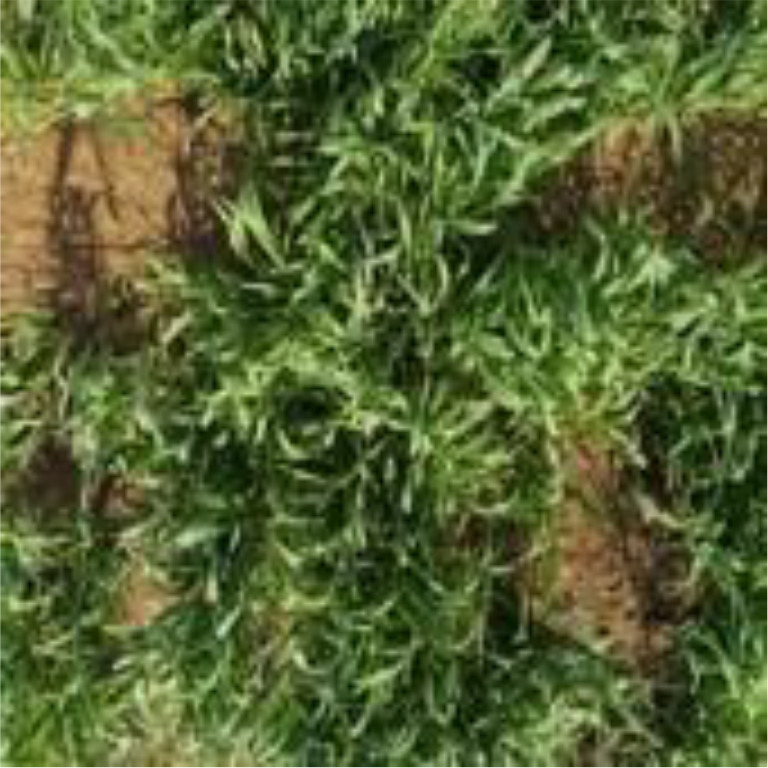	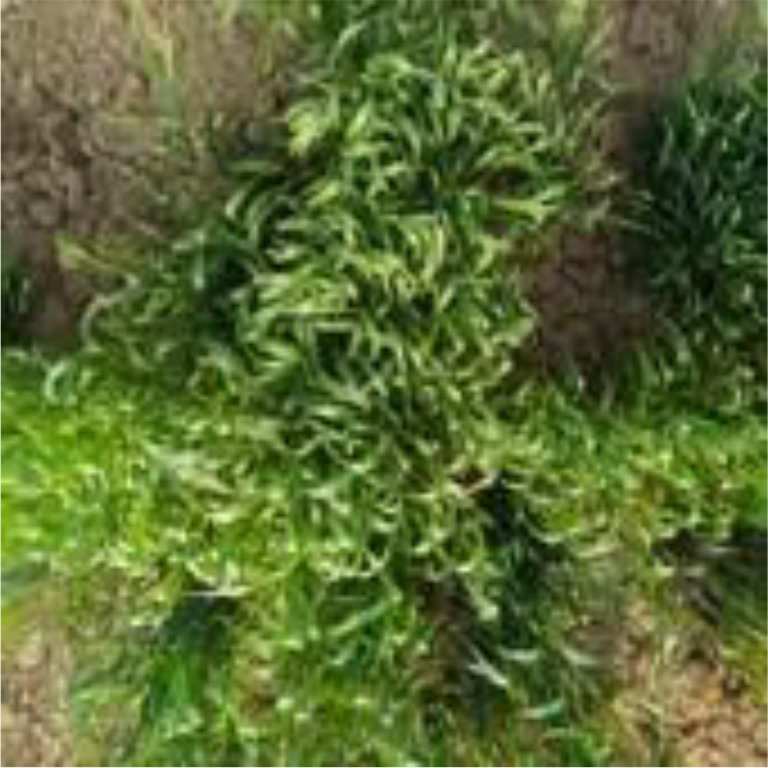
	H	34(May.17) 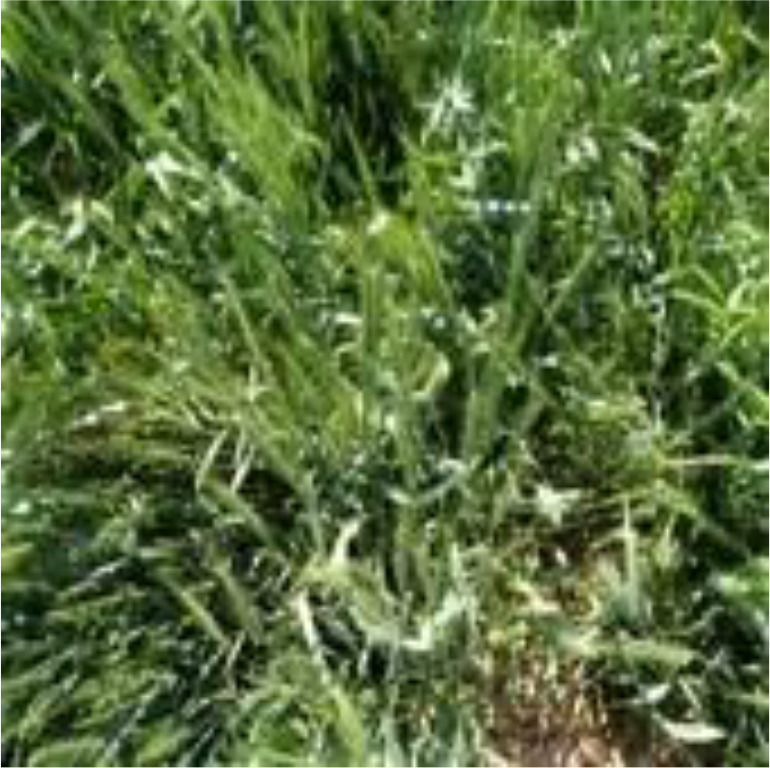	37(May.20) 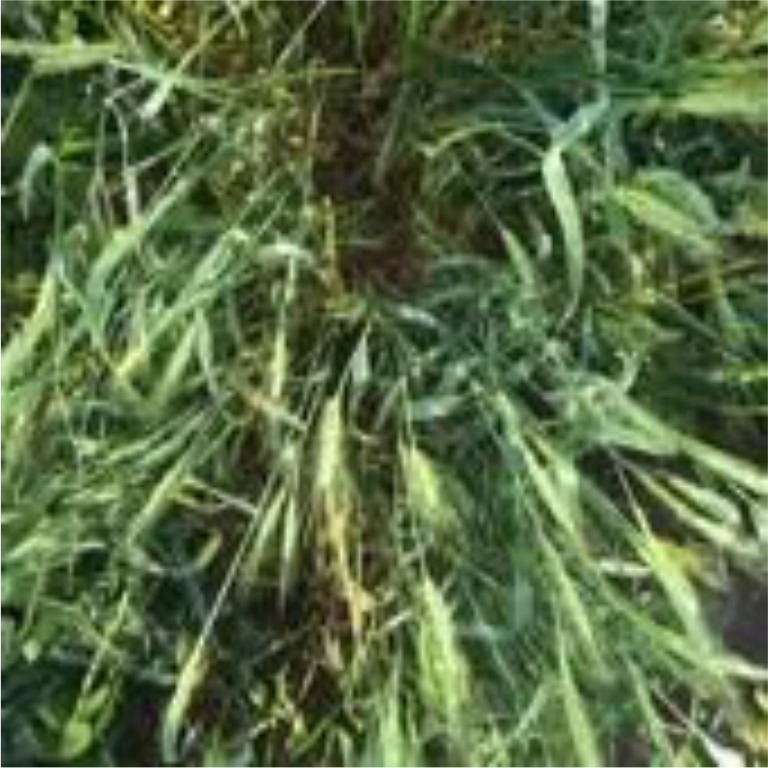	41(May.25) 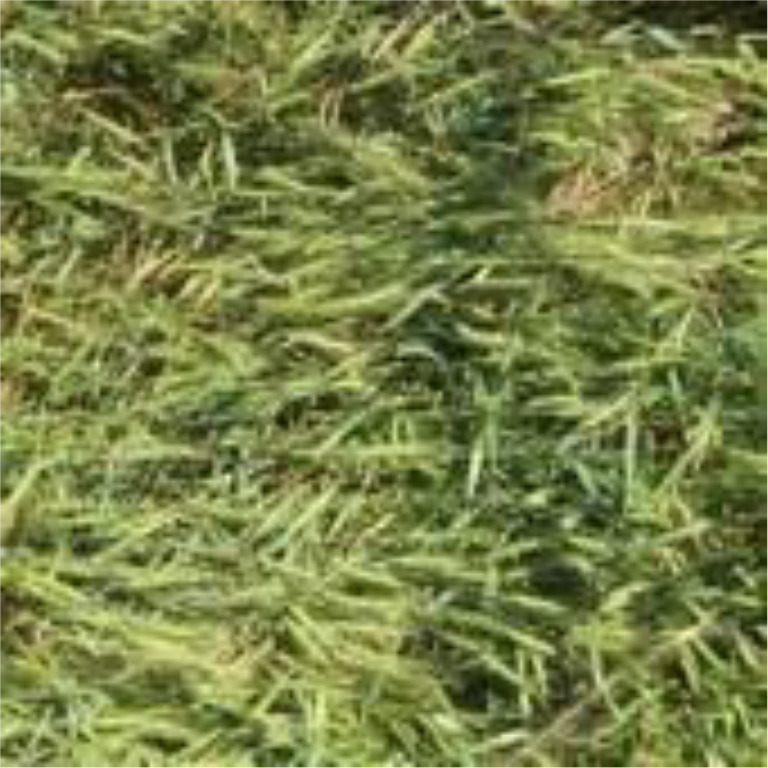	
	YR	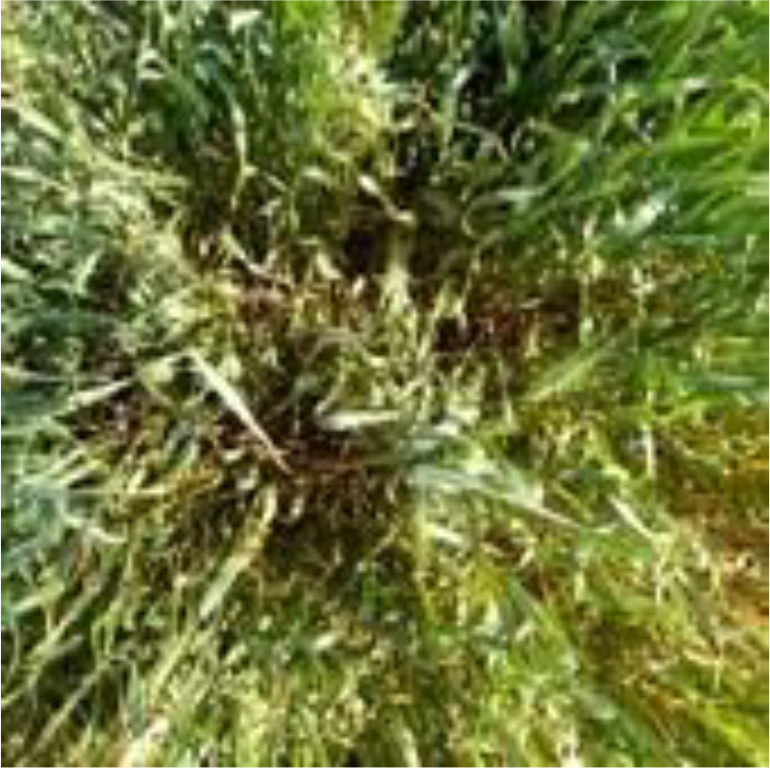	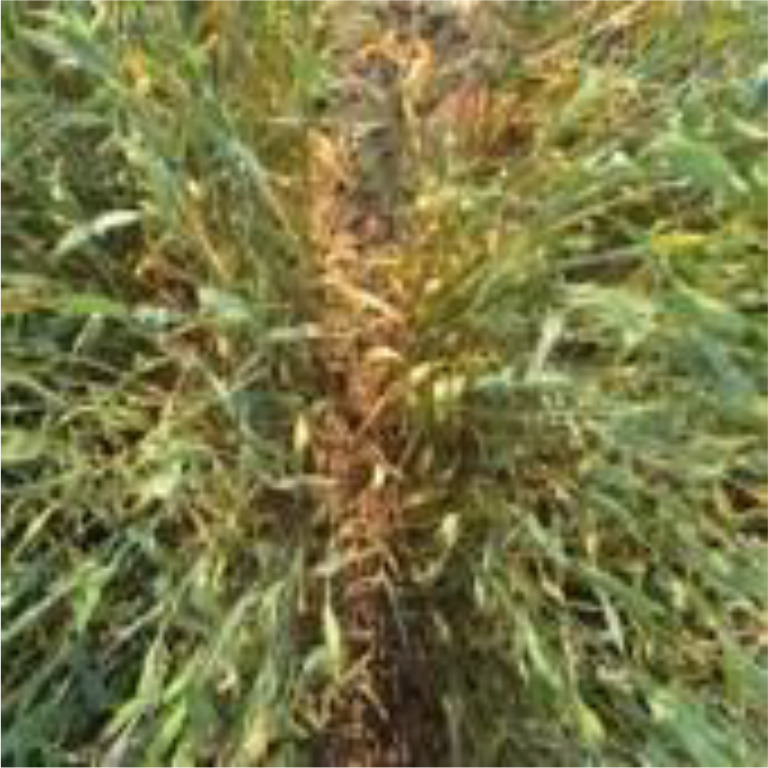	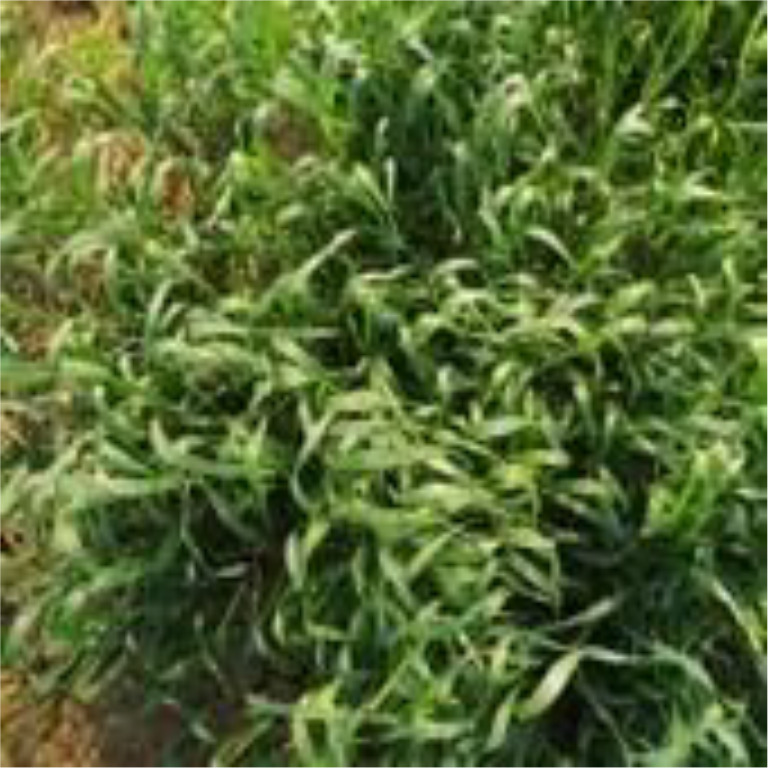	
Langfang 2018	H	7(Apr.18) 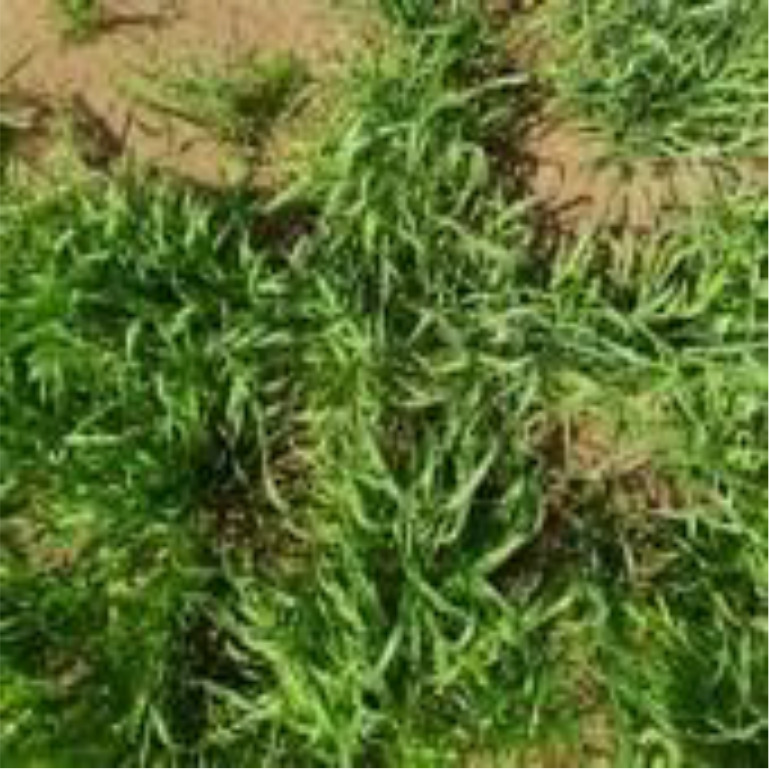	14(Apr.25) 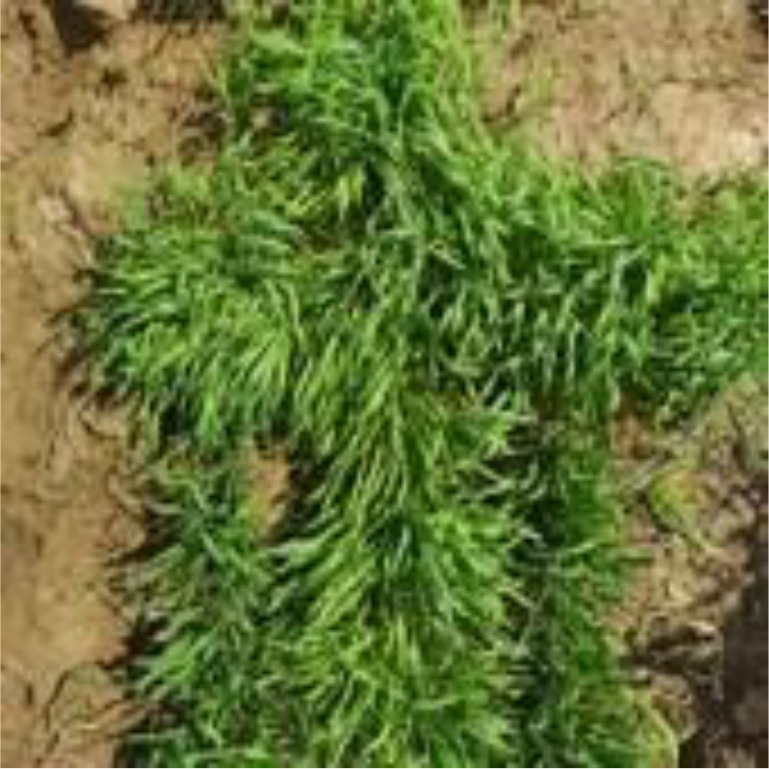	23 (May.4) 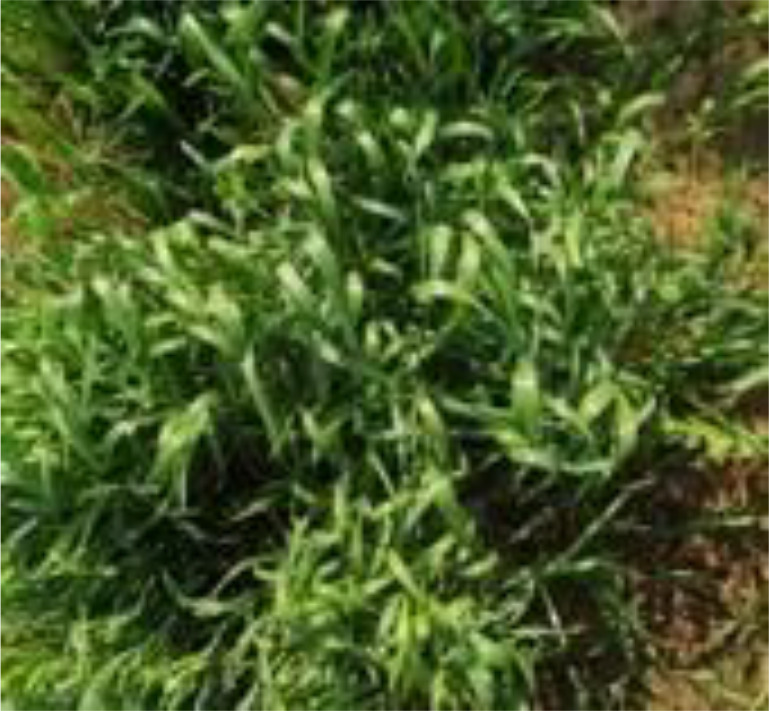	27(May.8) 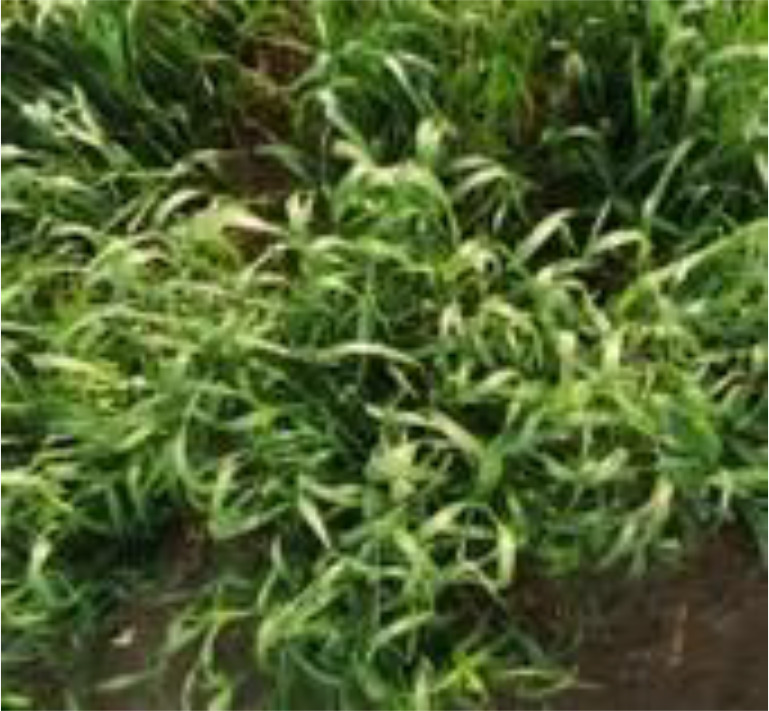
	YR	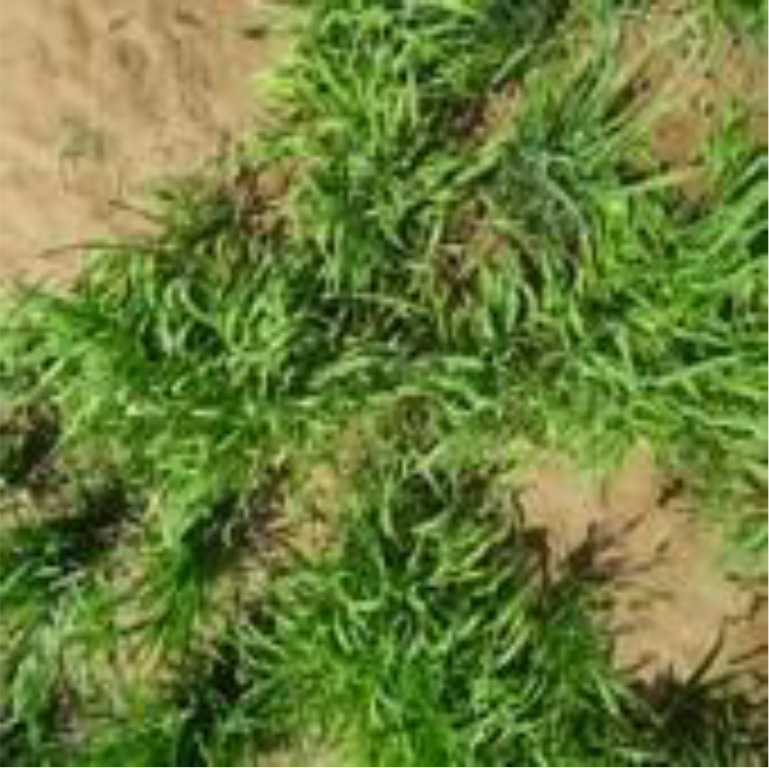	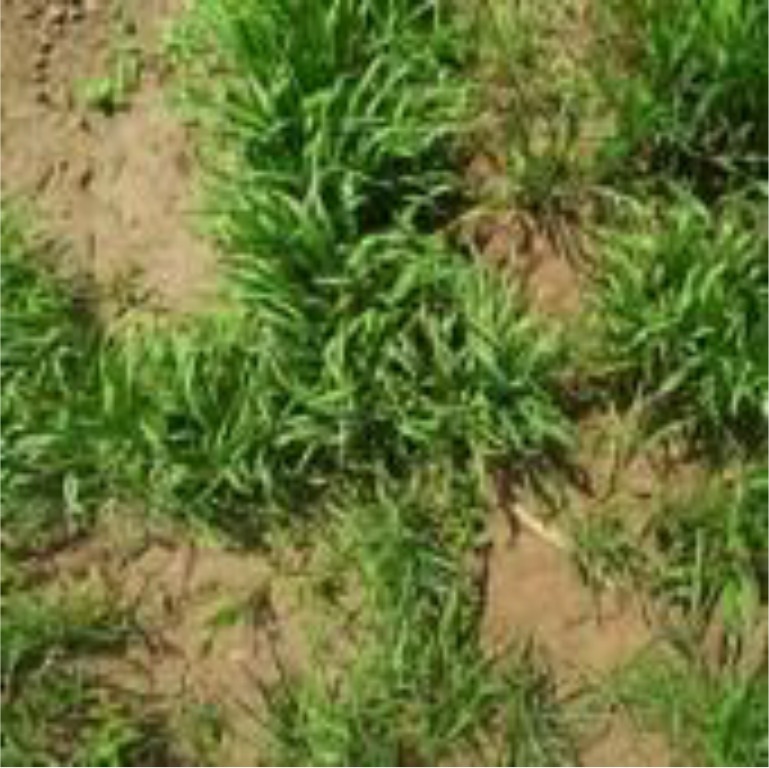	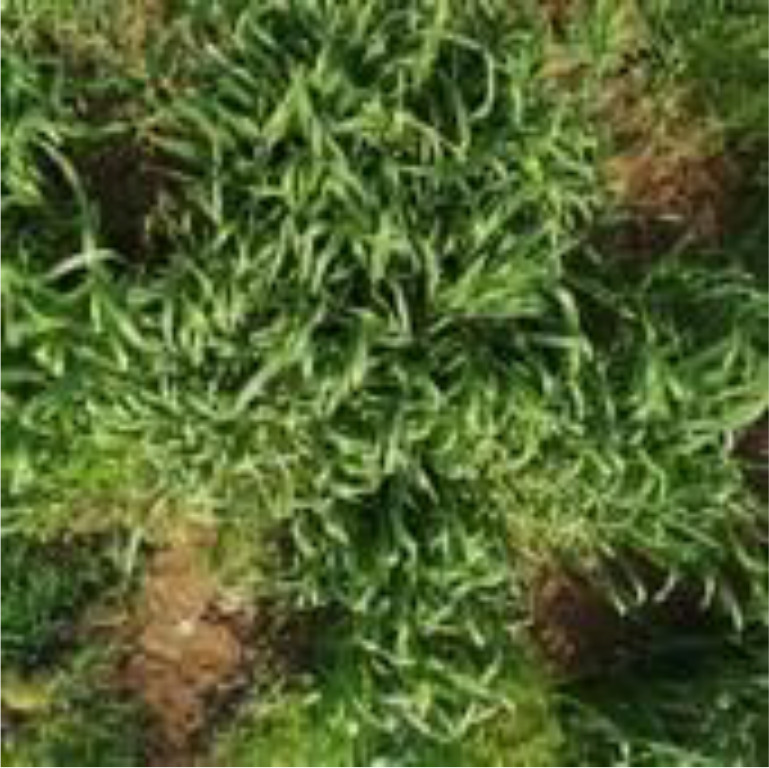	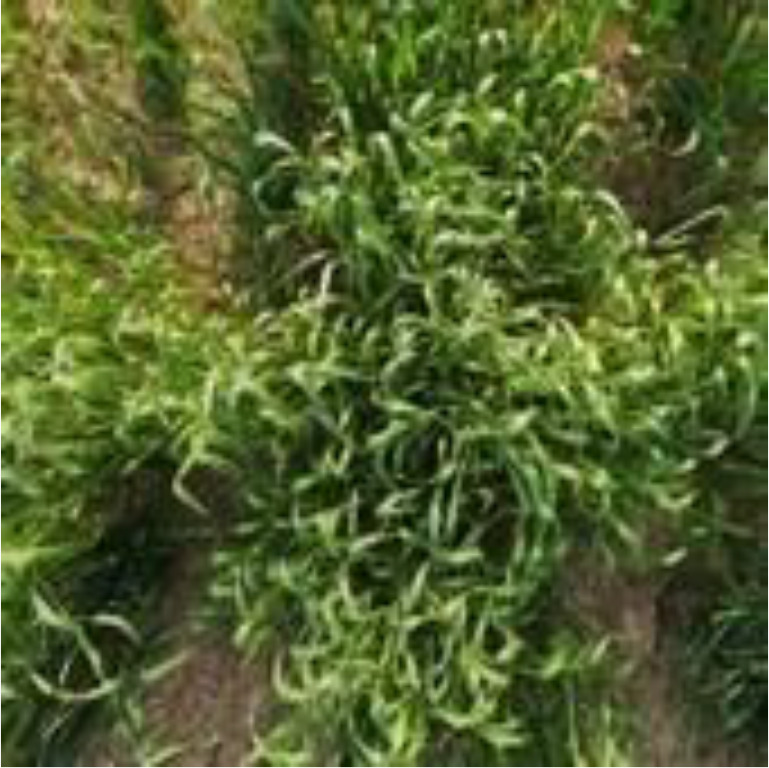
	H	34(May.15) 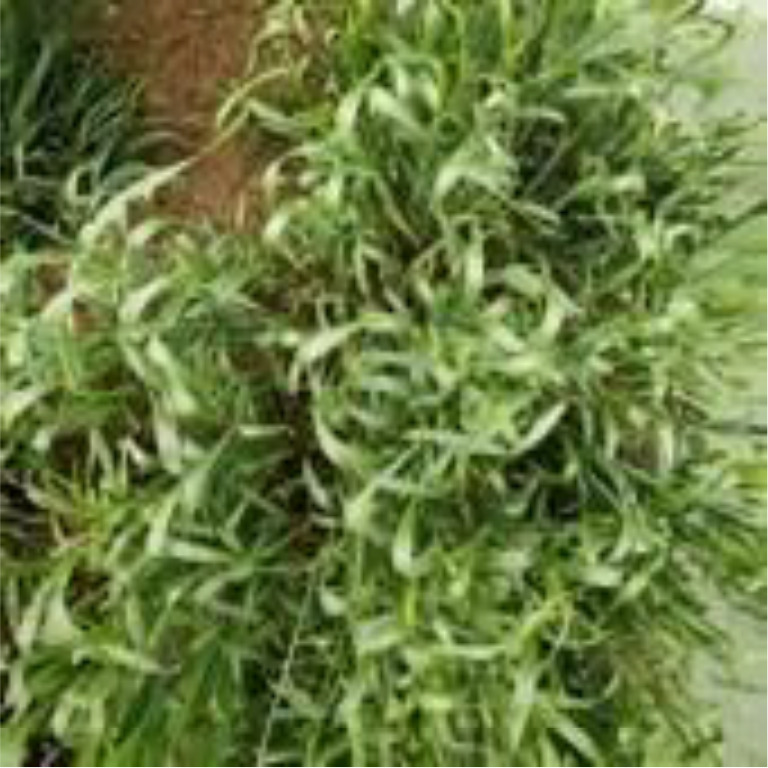	37(May.18) 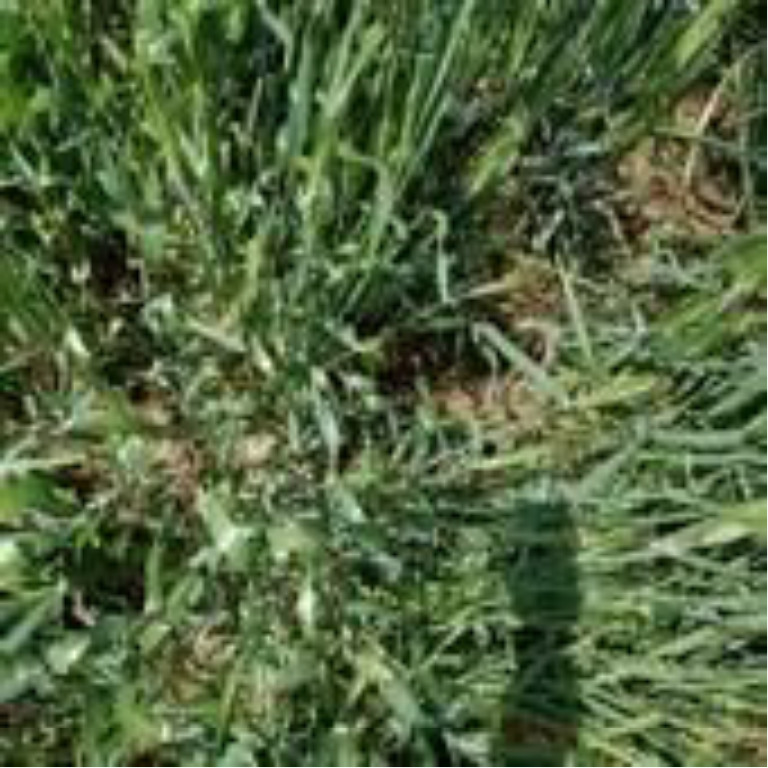	41(May.22) 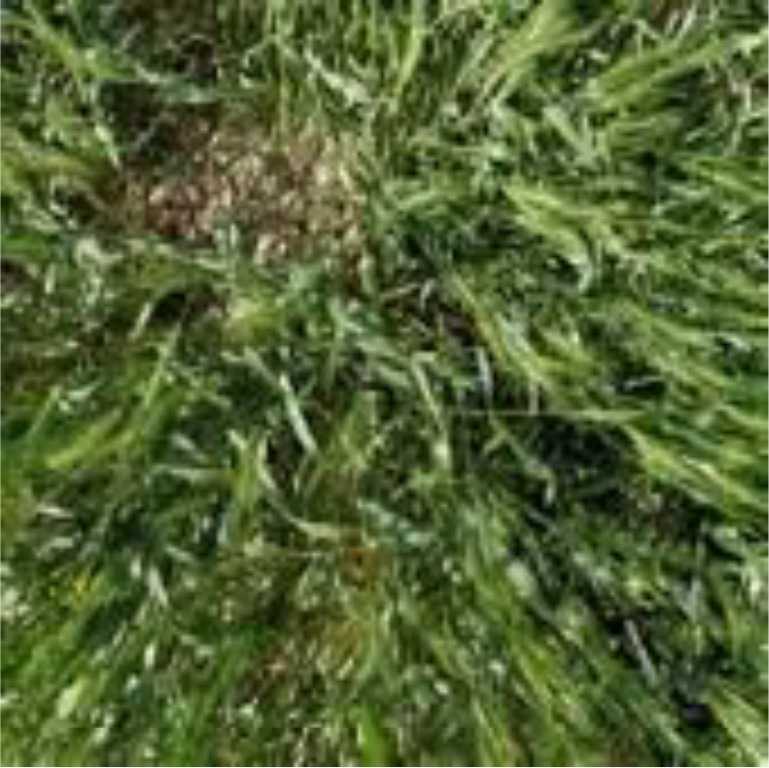	49(May.30) 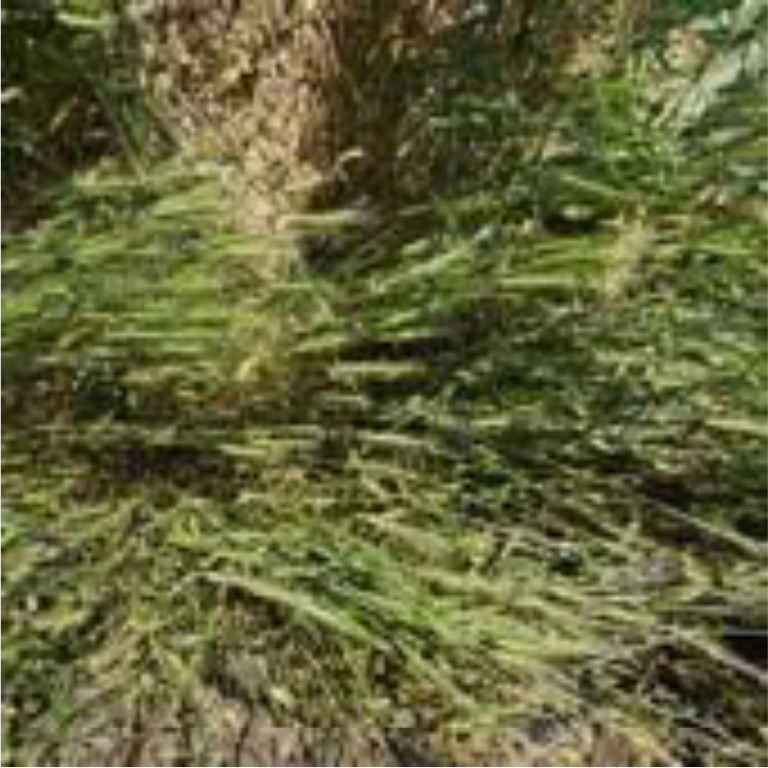
	YR	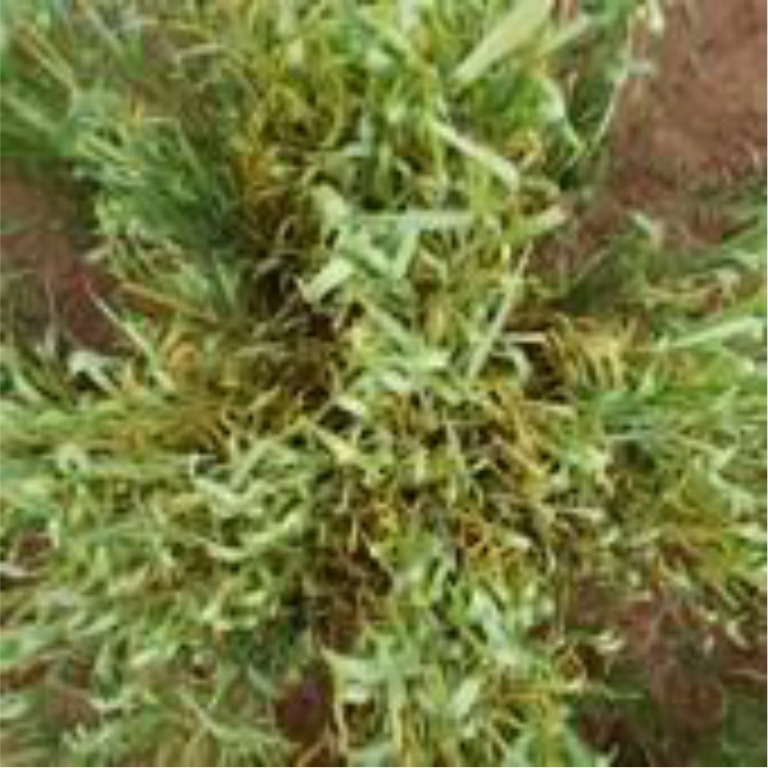	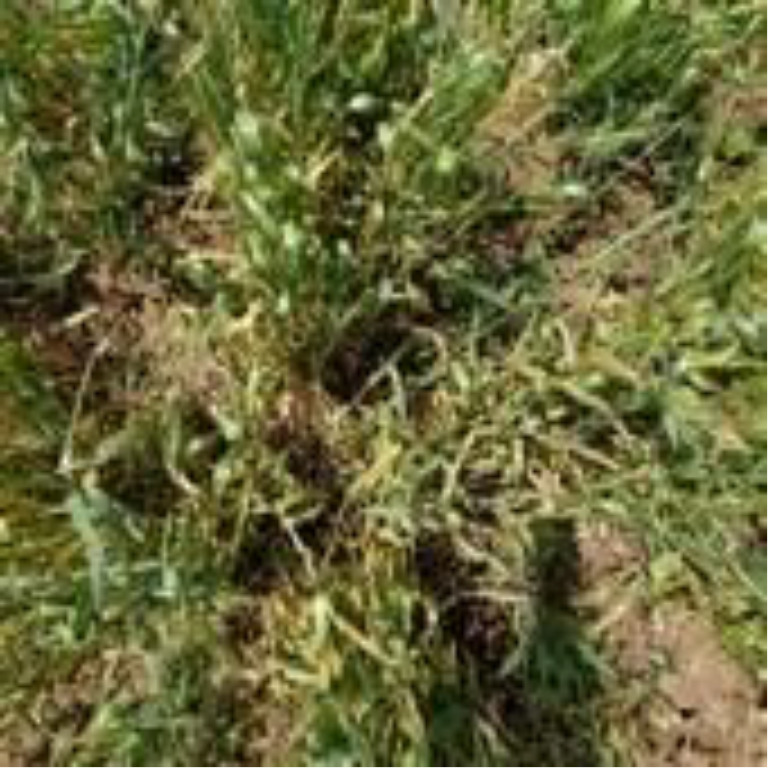	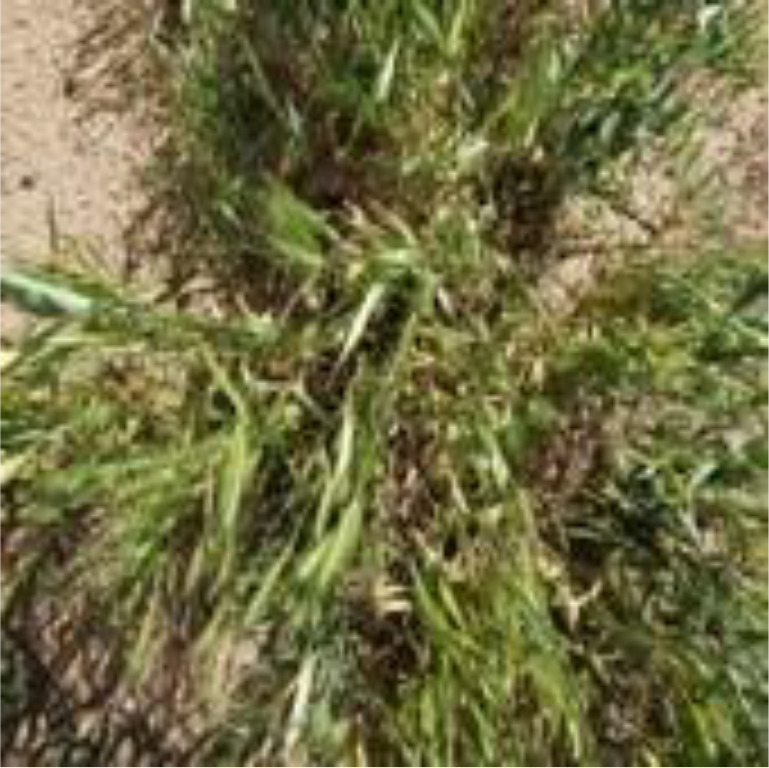	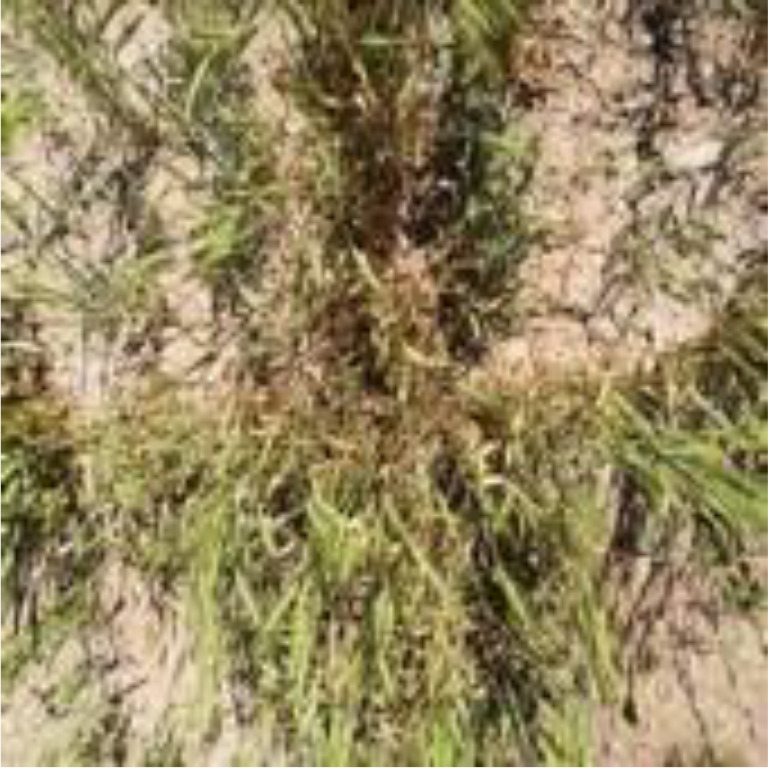
Xiaotang shan 2017	H	7(Apr.16) 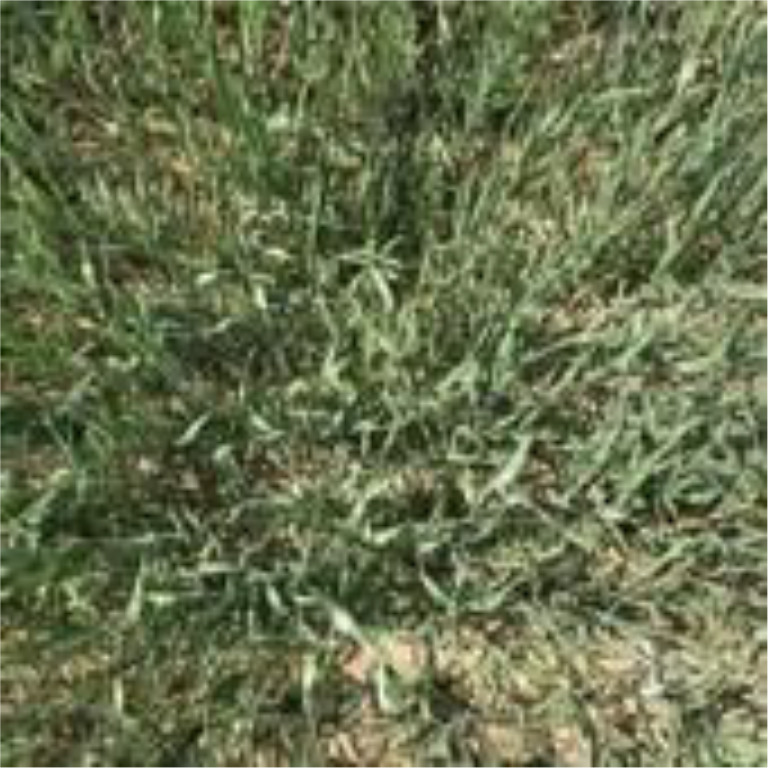	23(May.2) 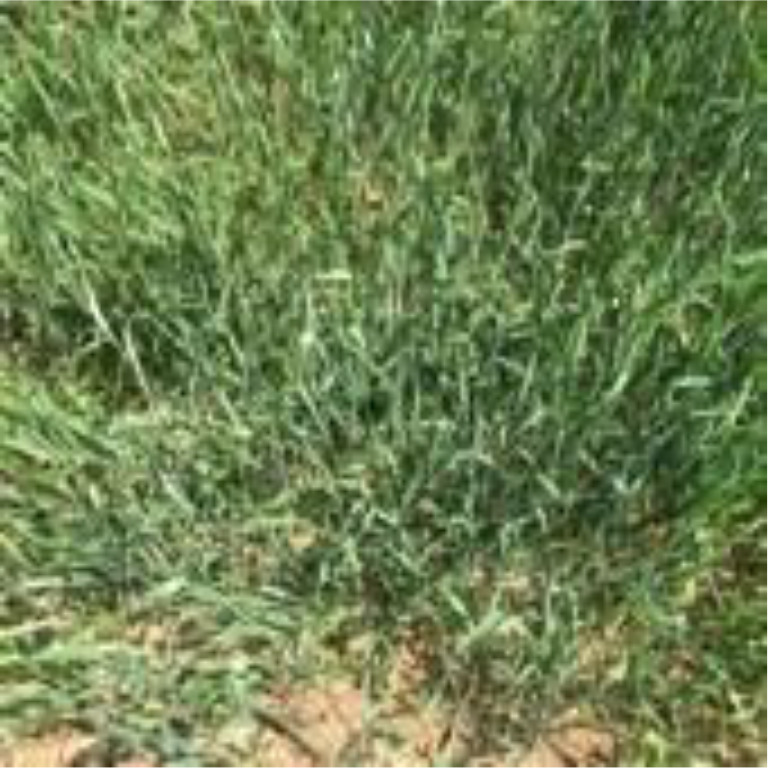	34(May.13) 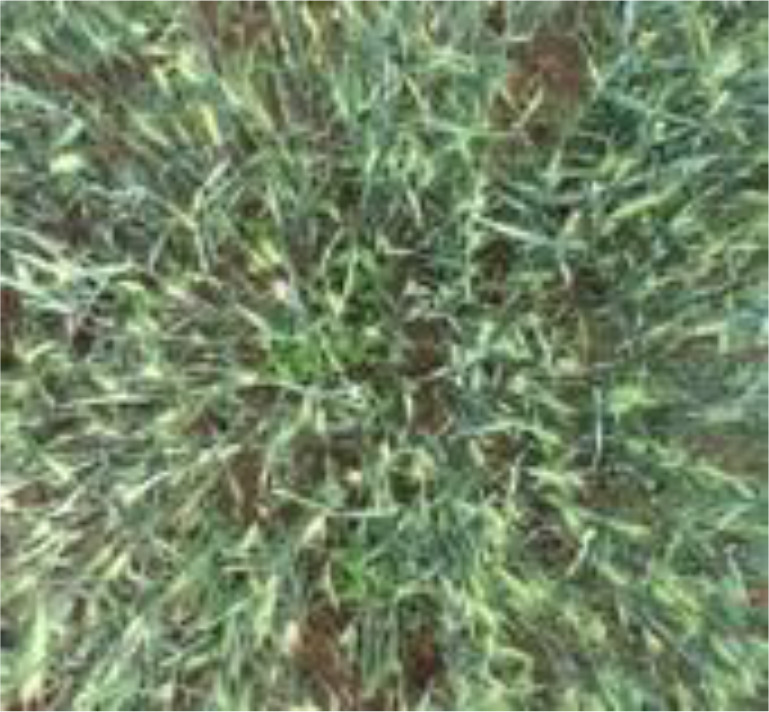	49(May.29) 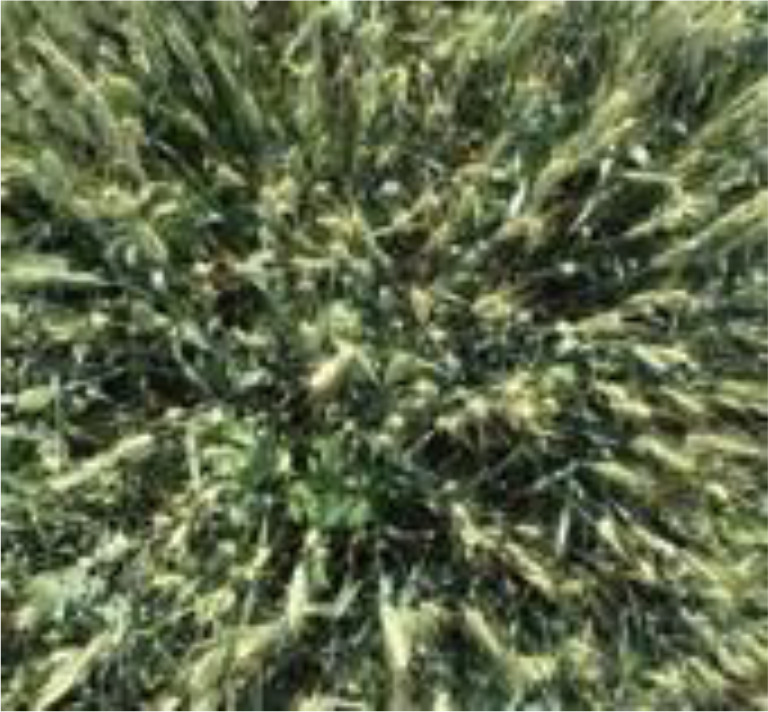
	ND	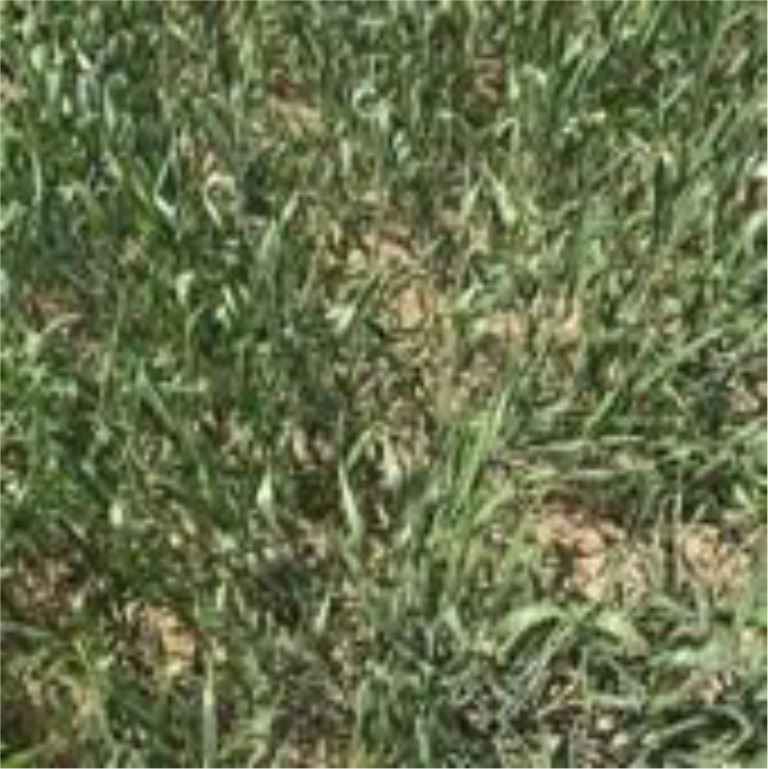	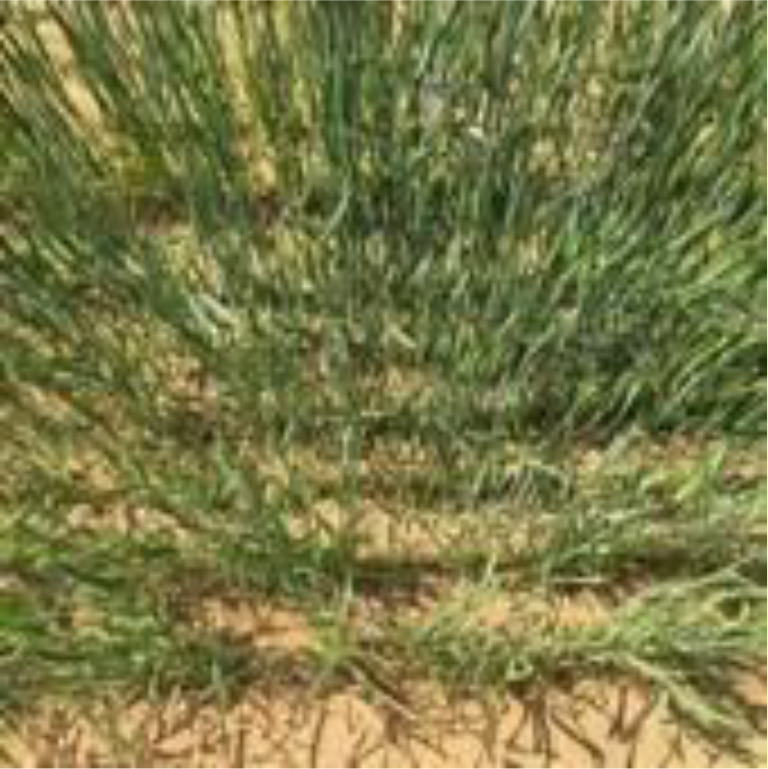	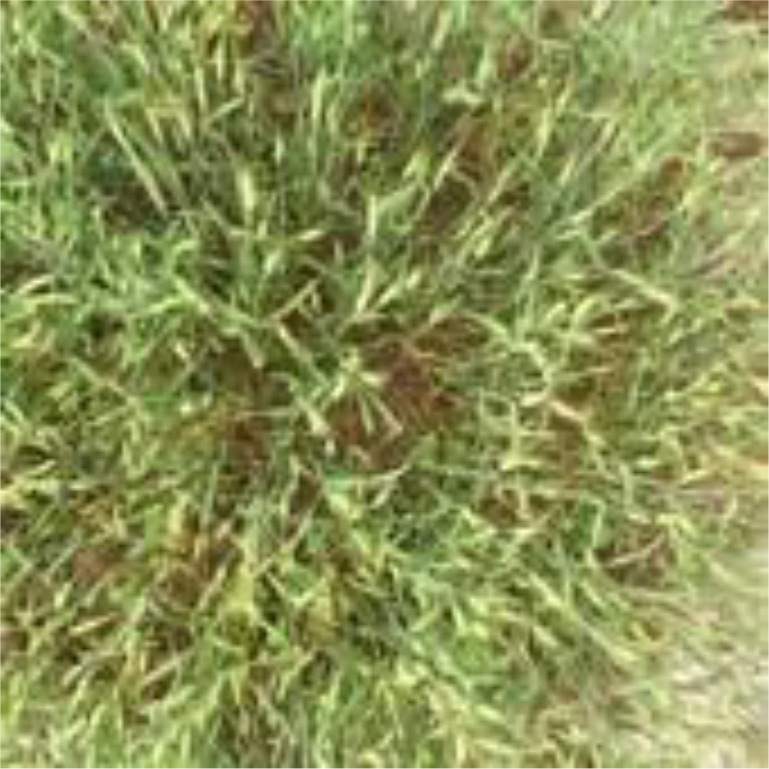	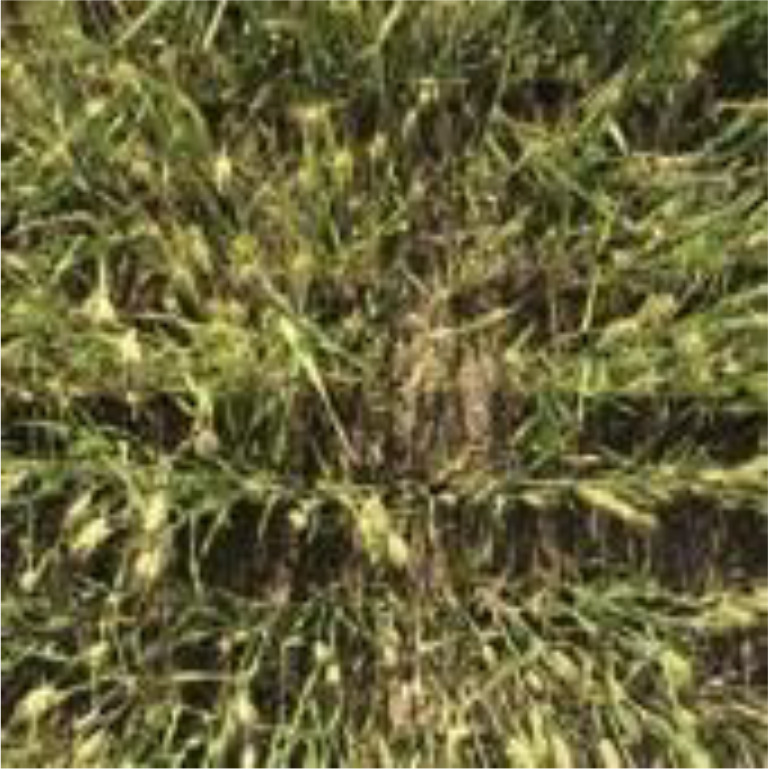
Xiaotang shan 2018	H	7(Apr.17) 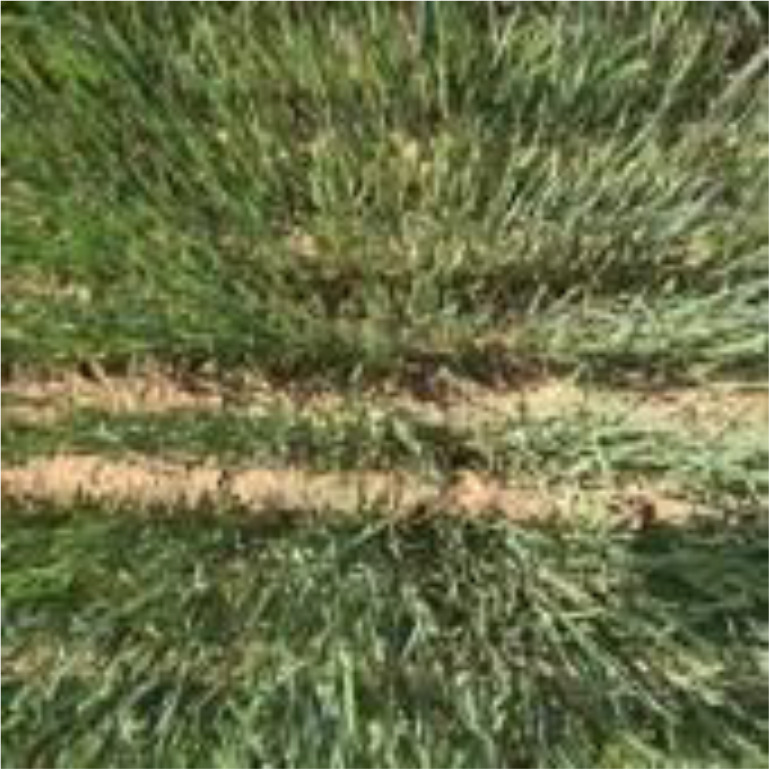	23(May.5) 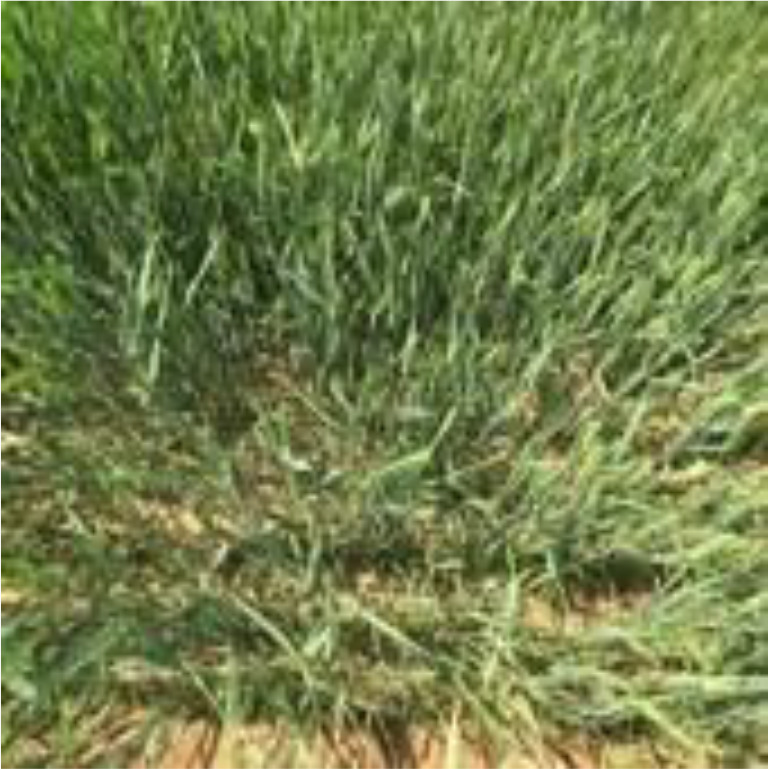	34(May.14) 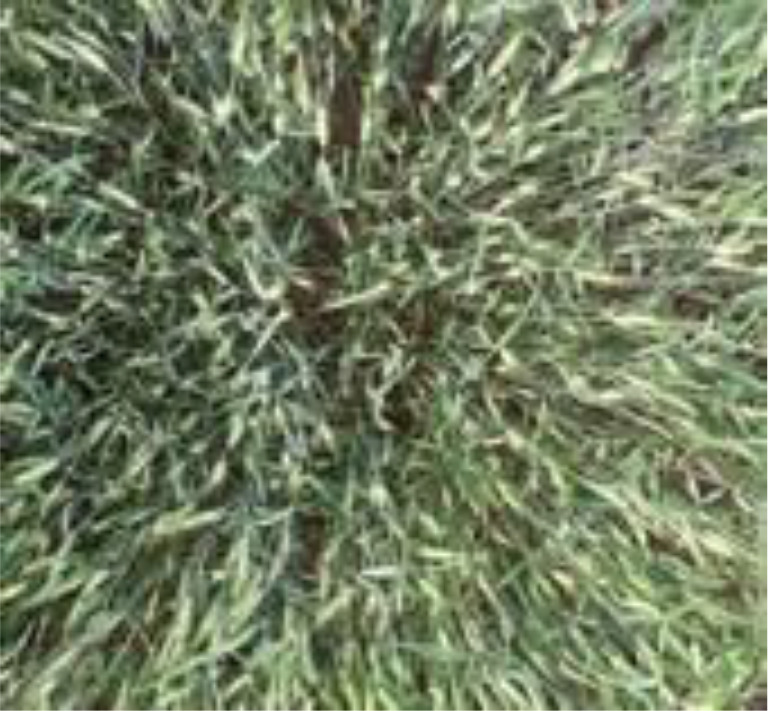	49(May.31) 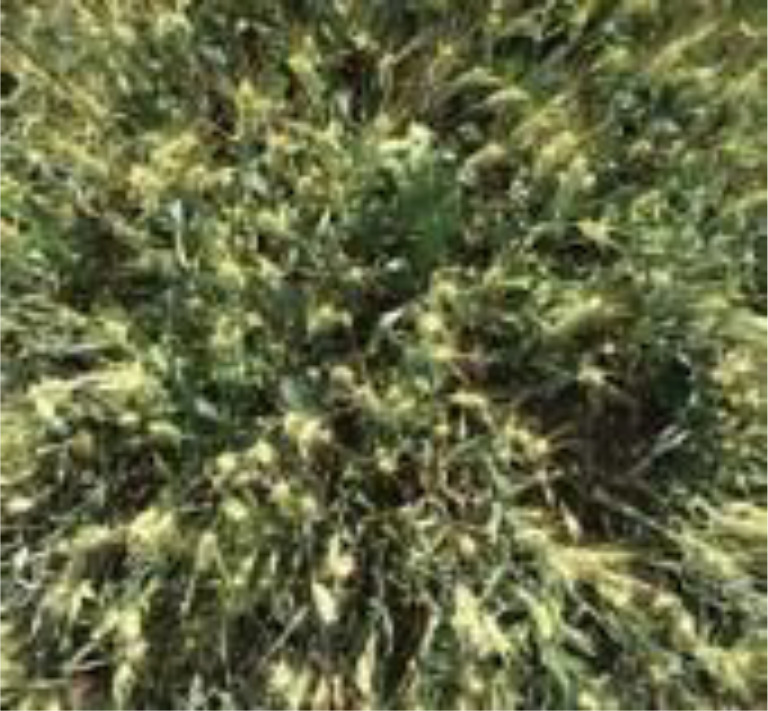
	ND	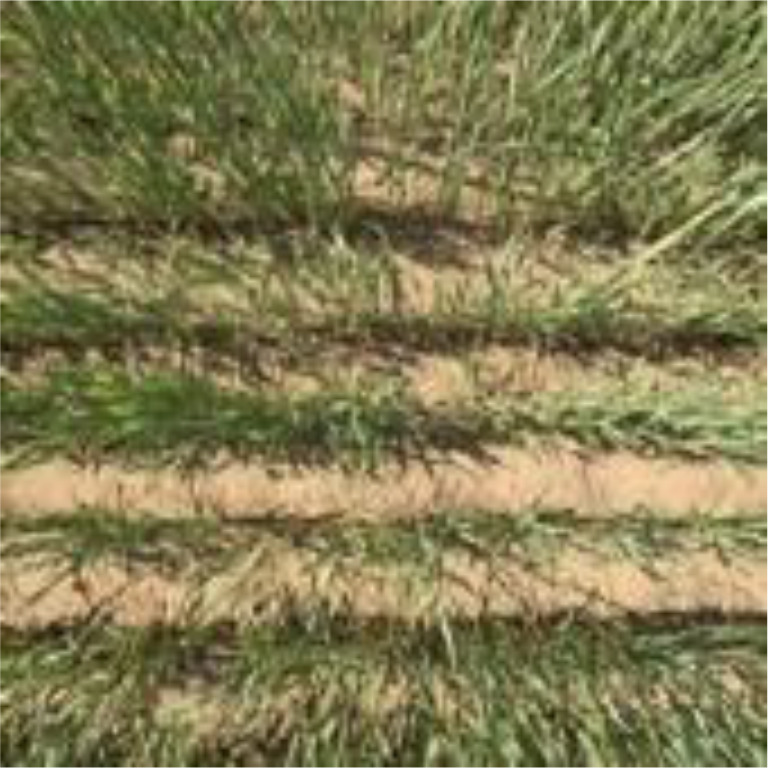	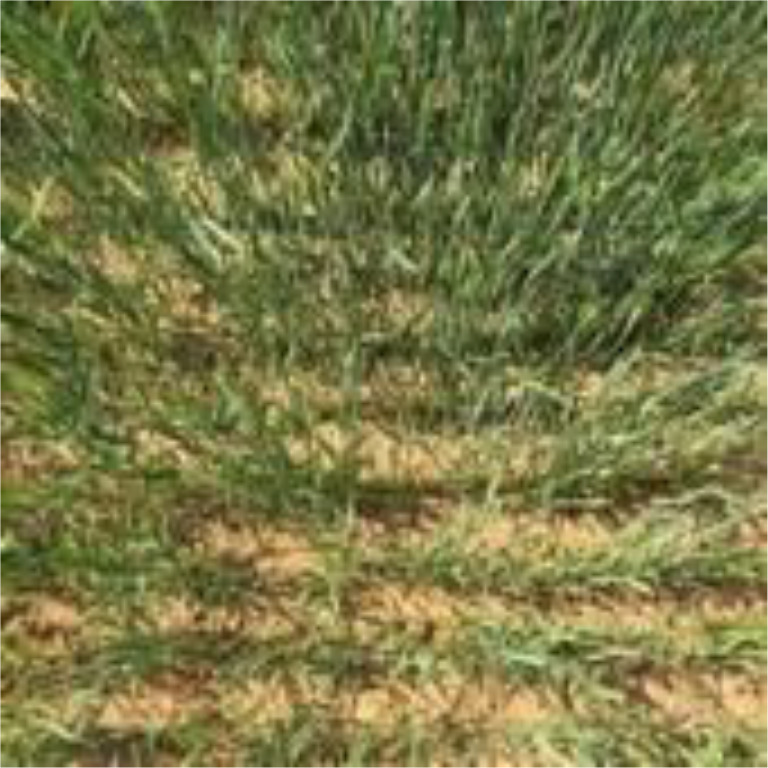	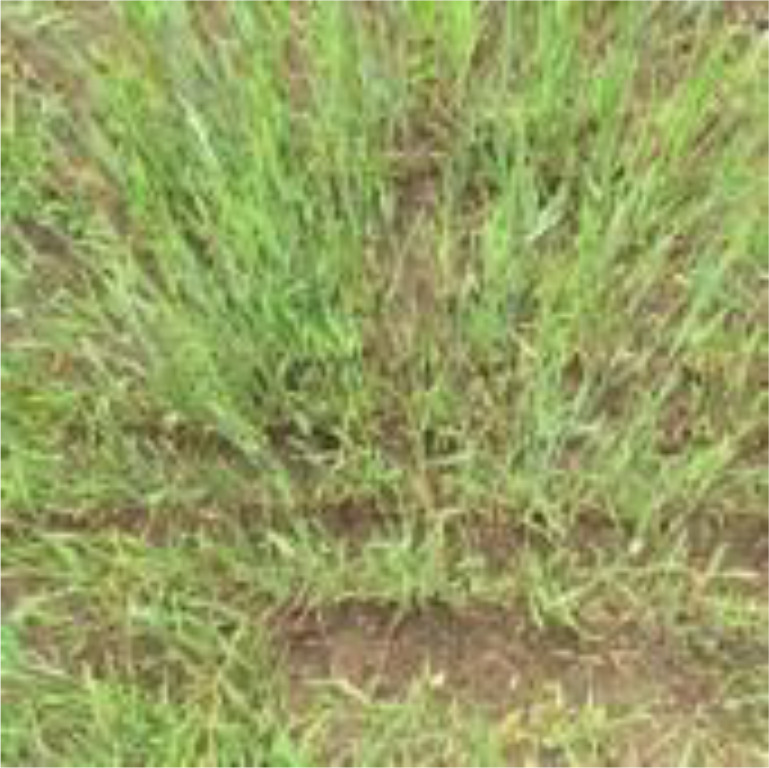	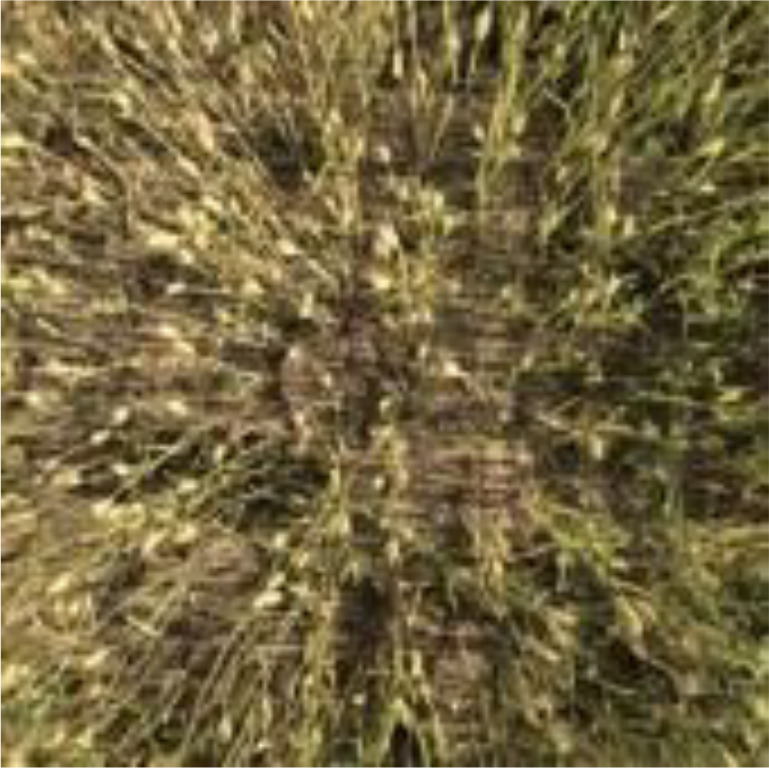

H, healthy; YR, yellow rust; ND, nitrogen deficiency.

For the yellow rust experiment, we used the wheat cultivar ‘Mingxian 169’ due to its susceptibility to yellow rust infestation. There was a control group and two infected groups of yellow rust (two replicates of inoculated treatment). Each field group occupied 220 m^2^ of field campaigns in which there were eight planting rows. For the control group, a total of eight plots (one plot in each row) with an area of 1 m^2^ were symmetrically selected in the field for hyperspectral observations and biophysical measurements. For the disease groups, the concentration levels of 5 mg 100^–1^mL^–1^ and 9 mg 100^–1^mL^–1^ spore solution were implemented to generate a gradient in infestation levels; eight plots were applied for sampling in each replicate. All treatments applied 200 kg ha^–1^ nitrogen and 450 m^3^ ha^–1^ water at the beginning of planting.

For the nitrogen deficiency experiment in Changping, the popular wheat cultivars ‘Jingdong 18’ and ‘Lunxuan 167’ were selected. There were two replicate field groups with the same nitrogen treatment applied. Each field group occupied 600 m^2^ of field campaigns in which three fertilization levels were used in 21 planting rows of field land (seven rows per treatment) at the beginning of planting, 0 kg ha^–1^ nitrogen (deficiency group), 100 kg ha^–1^ nitrogen (deficiency group), and 200 kg ha^–1^ nitrogen (control group). Similarly to Langfang, all treatments received 450 m^3^ ha^–1^ water at planting.

### 4.2 The simulation of Sentinel-2 bands

The simulated Sentinel-2 bands are regarded as the pure spectral signatures without the effects of atmosphere conditions. For this purpose, the reflectance and transmittances of the sampling plots were firstly collected using an ASD FieldSpec spectroradiometer (Analytical Spectral Devices, Inc., Boulder, CO, USA). In each plot, 10 scans were taken at 1.2 m above the wheat canopy. The spectroradiometer was fitted with a 25° field-of-view bare fiber-optic cable and operated in the 350-nm–2,500-nm spectral region. The sampling interval was 1.4 nm between 350 nm and 1,050 nm and 2 nm between 1,050 nm and 2,500 nm. A white spectral reference panel (99% reflectance) was acquired once every 10 measurements to minimize the effect of possible differences in illumination. Only the bands in the range of 400 nm–1,000 nm were adopted in this study in order to match the visible-red edge-near infrared bands of Sentinel-2 and avoid bands below 400 nm and above 1,000 nm that were affected by noises (Shi et al., 2017b). In order to keep radiance consistence, the sampling was conducted at the same period of time between 11:00 and 13:30 local time under a cloud-free sky.

Subsequently, we integrated the field canopy hyperspectral data with the sensor’s relative spectral response (RSR) function to simulate the multispectral bands of Sentinel-2. The formula is given as follows:


(6) $$R_{sentinel-2}=\frac{\int_{\text{λ}start}^{\text{λ}end}R_{ground}(\text{λ})\cdot RSR(\text{λ})dx}{\int_{\text{λ}start}^{\text{λ}end}RSR(\text{λ})dx}$$

where *R_sentinel–_*_2_ is the simulated multispectral channel of Sentinel-2 sensor; λ*_start_* and λ*_end_* represent the beginning and ending reflectance wavelength of Sentinel-2’s corresponding channel, respectively; *R_ground_* is the ground truth canopy hyperspectral data; and RSR is the relative spectral response of Sentinel-2 sensor (https://earth.esa.int/web/sentinel/user-guides/sentinel-2-msi/document-library/). Both the *R_ground_* and RSR are the functions of wavelength.

### 4.3 Collection of ground truth plant parameters

The plant LAI and LCC were synchronously measured on the same place where the canopy spectral measurements were made. The LCC was measured by the Dualex Scientific sensor (FORCE-A, Inc., Orsay, France), a handheld leaf-clip sensor designed to nondestructively evaluate the content of chlorophyll and epidermal flavonols. The LCC values were collected with the default unit, which were used preferentially because of the strong relationship between their digital readings and real foliar chlorophyll. Considering the canopy structure-derived multiple scattering process, the first three leaves from the top are regarded as the most effective one with maximum photosynthetic absorption rate, which not only represent the average growth state of the whole plant but also contribute most to the canopy reflected radiation measured by our observations. Therefore, for each sampling plot, the first, second, and third wheat leaves, from the top of 10 randomly selected plants (30 leaves for each plot), were chosen for LCC measurements. For the LAI acquisition, the LAI-2200 Plant canopy analyzer (Li-Cor Biosciences Inc., Lincoln, NE, USA) was used in each 1 m × 1 m subplot.

### 4.4 Assessment of ground truth plant stress severity

**Figure 3:**
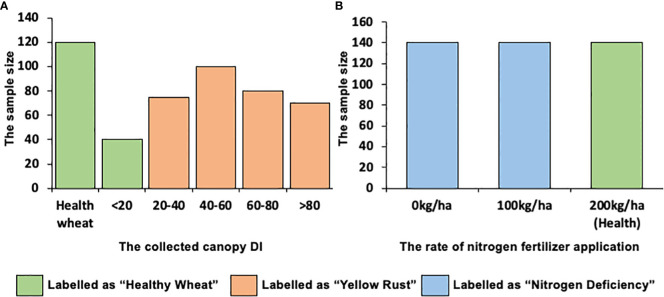
The distribution of the **(A)** collected disease index (DI) of Yellow Rust and **(B)** fertilization levels of Nitrogen Deficiency.

In this study, the disease index (DI) was used to measure the severity of yellow rust, and the fertilization level was used to measure the severity of nitrogen deficiency. Specifically, the DI was calculated using the method mentioned in Zhang et al. (2012). It is noted that because slight stress (DI< 20) generates an invisible influence on wheat yield and does not trigger enough spectral responses on the top-of-canopy (TOC) reflections of the 10 m × 10 m Sentinel-2 pixels, the samples with DI< 20 were labeled as “healthy wheat”; otherwise, they were labeled as “yellow rust.” In order to guarantee the uniformed bias in each observation, all leaves were manually inspected by the same specially assigned investigators according to the National Rules for the Investigation and Forecasting of Plant Diseases (GB/T 15795-1995). For nitrogen deficiency, three fertilization levels (i.e., 0 kg ha^–1^, 100 kg ha^–1^, and 200 kg ha^–1^) were controlled in our experiments; here, we labeled the fertilization level of 200 kg ha^–1^ as “healthy wheat”; otherwise, they were labeled as “nitrogen deficiency.” The distribution of the collected DI of yellow rust and the fertilization levels of nitrogen deficiency is shown in **Figure 3**.

### 4.5 The ground survey dataset under natural field conditions

**Figure 4:**
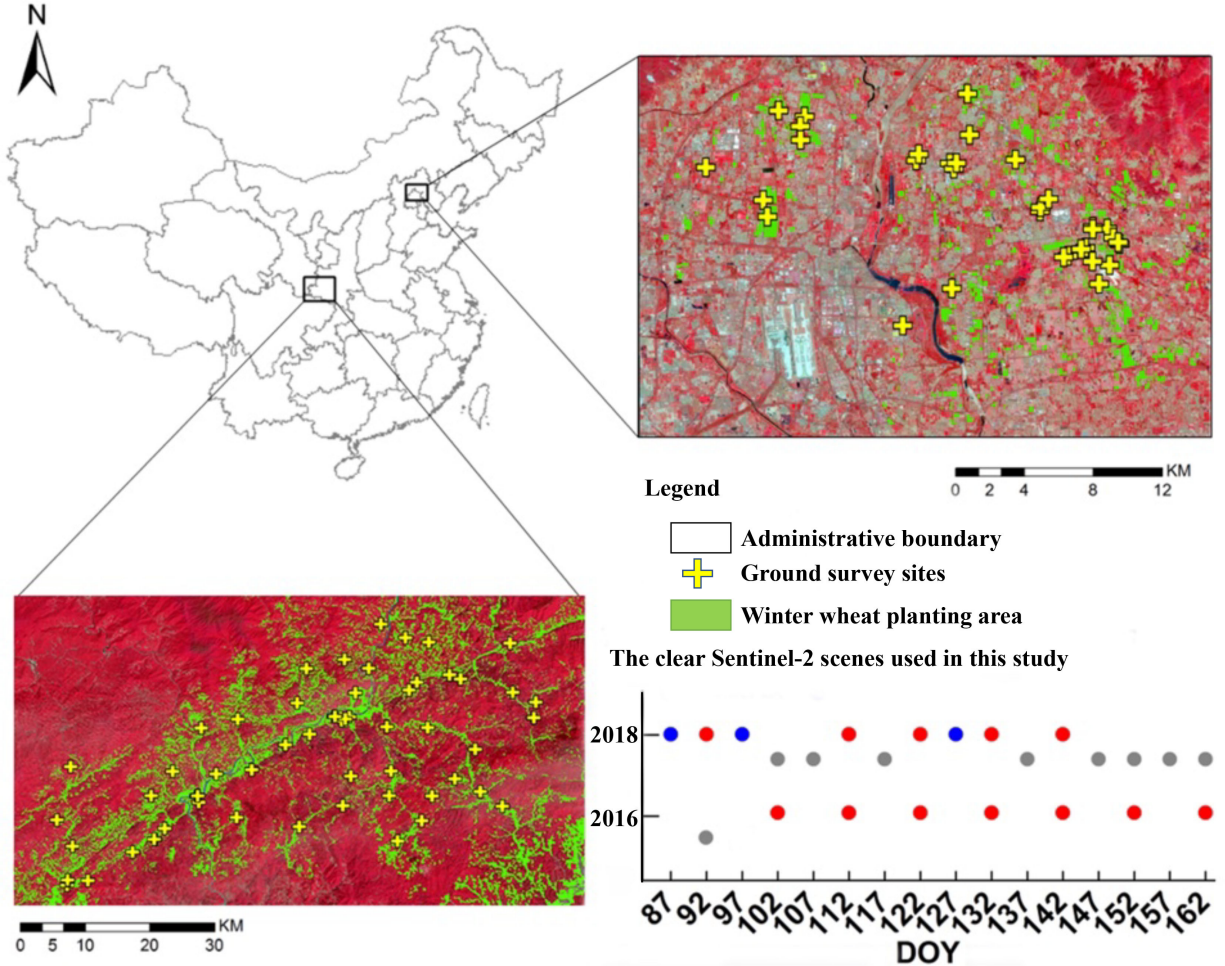
False-color maps of the experimental sites of Ningqiang county, Shaanxi (bottom left), and Shunyi district, Beijing (top right). Overview of the Sentinel-2 imagery used.

In order to evaluate the generalization and transferability of the proposed model in actual applications under natural conditions, we collected the actual Sentinel-2 time series and the ground truth data in two different sites, one is located in the Ningqiang county (37°35′51″N, 118°35′19″E), Shaanxi province, 2018, and another one is located in Shunyi district (41°20′41″N, 116°24′8″E), Beijing, 2016. In Ningqiang county, a total of nine cloud-free Sentinel-2 images and 55 ground truth plots were collected. In Shunyi district, a total of six cloud-free Sentinel-2 images and 32 ground truth plots were collected. All of the collected Sentinel-2 images were atmospherically corrected using the SEN2COR procedure, converting top-of-atmosphere (TOA) reflectance into TOC reflectance. TOC products were the result of a resampling procedure with a constant ground resampling distance of 10 m for visible and near-infrared bands (B2, B3, B4, and B8) and 20 m for red-edge bands (B5, B6, B7). The spatial resolution of the red-edge bands (B5, B6, B7) was homogenized to 10 m using nearest neighbor resampling. Such process was conducted in the ESA SNAP 6.0 software. The basic principle of the nearest neighbor resampling was described in the study by Roy and Yan (2020). The overview of the sampling plots and Sentinel-2 collection is shown in **Figure 4**.

**Figure 5:**
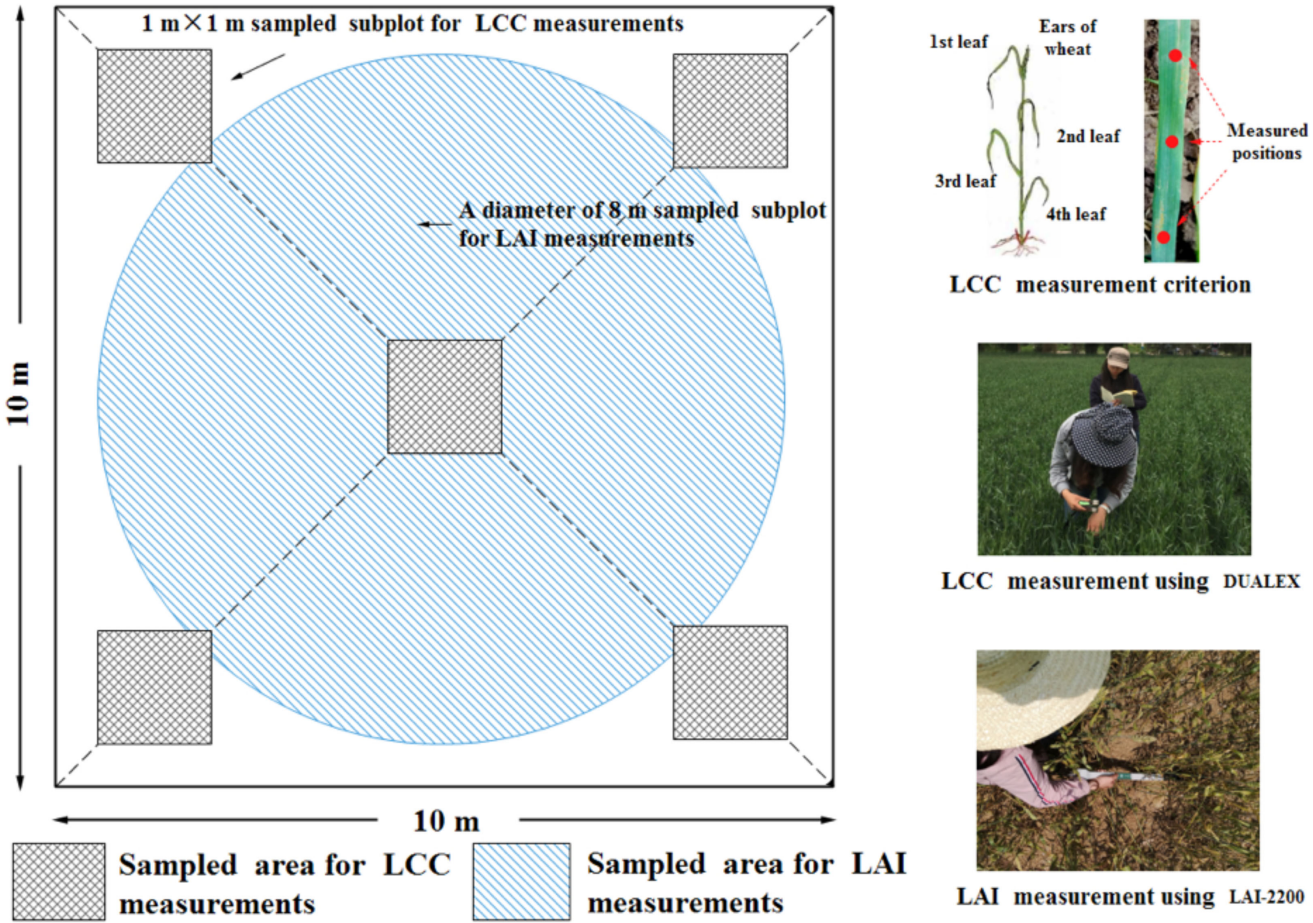
The measurement sketch of the synchronously ground LAI and LCC truth data collection.

In both surveys, LAI and LCC values were measured by the same approaches used in the experiments under controlled field conditions. Each sample was collected in an area of approximately 10 m × 10 m (corresponding to the spatial resolution of Sentinel-2 bands), of which the center coordinates were recorded using a GPS with differential correction (accuracy in the order of 2–5 m). The sketch of the sampled site setting is shown in **Figure 5**.

**Figure 6:**
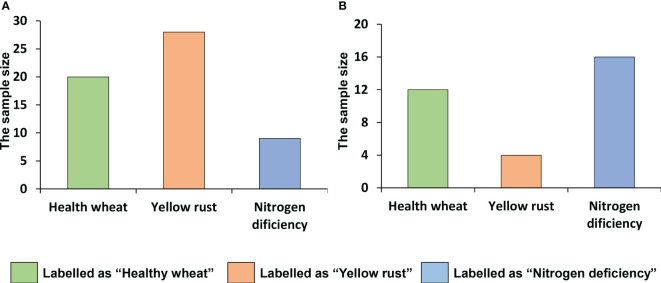
The distribution of the labeled classes in **(A)** Ningqiang and **(B)** Shunyi.

DIs of yellow rust were measured by the same method used in the experiments under controlled field conditions. In each plot, a plot was labeled as “yellow rust” when DI > 20. On the other hand, nitrogen deficiency in each plot was investigated by requesting the history of fertilizer application to the local farmers, and a plot was labeled as “nitrogen deficiency” when the history of fertilizer application was < 150 kg/ha. The statistical distribution of the labeled classes was shown in **Figure 6**.

## 5 Results and discussion

In this section, the proposed model is tested and evaluated in three different aspects, including the model performance on detecting and discriminating the yellow rust and nitrogen deficiency, computing efficiency and robustness, and the interpretability assessment.

Firstly, to test the performance of the proposed FFCDNN on detection and discrimination of yellow rust and nitrogen deficiency, three representative methods, including BIT-DNN (Shi et al., 2021), which represents the state-of-the-art interpretable learning model, AlexNet (Lv et al., 2020), which represents the advanced deep learning model on remote sensing objective detection, and support vector machine (SVM), which represents the typical machine learning method. Specifically, for CapsNet, the network architecture is proposed in Shi et al. (2021). For AlexNet, the network architecture and hyperparameter setting is referred to Han et al. (2017). For the configuration of the SVM classifier, the radial basis function (RBF) kernel is used in the SVM classification frame, and a grid-based approach proposed by Rumpf et al. (2010) is used to specify the parameter *C* and.

Regarding the model assessments, six evaluation metrics, including F1 score, average accuracy, producer’s accuracy, user’s accuracy, Kappa value, and computing time, are employed in this study to evaluate the classification accuracy and robustness. The definitions of these matrices are formulated in Mahlein et al. (2017) and Lv et al. (2020).

Secondly, for the interpretability assessment of the model, a *post-hoc* analysis is used to expose the learning process and feature representations of the data life in the proposed model. Specifically, a canonical discriminant analysis is first used to measure the intra-class distance and the separability in each learning stage of the model. The definition of the canonical discriminant analysis is described in our previous study (Shi et al., 2017a). And then, the coefficients of determination (*R* ^2^) between the generated biologically composed features and the ground-measured severity of yellow rust and nitrogen deficiency are calculated based on univariate correlation analysis.

### 5.1 Model test on detection and discrimination of the yellow rust and nitrogen deficiency

#### 5.1.1 Experiment 1: model testing on the simulated Sentinel-2 bands under controlled field conditions

The first experiment is to evaluate the performance of the proposed model on the detection and discrimination of yellow rust and nitrogen under controlled conditions. For model testing and validation, 5-fold cross-validation is employed. The comparison of the classifications of the proposed FFCDNN, BIT-DNN, AlexNet, and SVM is shown in **Table 2**. Our results show that for the model testing process, the proposed FFCDNN achieves >90% classification accuracy that was consistent with the performance of the baseline models. Nevertheless, for the model evaluation process, the proposed method achieves the best performance with 92.12% overall accuracy, 6.51% higher than the second best model (i.e., BIT-DNN). These findings suggest that the proposed model has great robustness and generalization for the plant stress detection and classification. In addition, it is of note that the misclassification mainly occurs between healthy wheat and nitrogen deficiency. In terms of computing efficiency, although the computing time of the proposed model is not the best among the baseline, it is highly improved from the convolution-based deep learning model.

**Table 2:** The assessment of the proposed model and the baseline models in terms of producer’s accuracy (PA), user’s accuracy (UA), F1 score (F1), overall accuracy (OA), Kappa, and computing time (CT).

Model	Class	PA(%)	Testing dataset			Kappa	CT(s)
			UA(%)	F1(%)	OA(%)		
FFCDNN	Health YR	97.74 95.15	97.34 96.21	97.54 95.68	95.13	0.891	277.4
	NS	91.98	92.35	92.16			
BIT-DNN	Health YR	92.72 93.51	94.25 93.55	93.48 93.53	92.07	0.881	299.8
	NS	88.86	89.54	89.2			
AlexNet	Health YR	90.61 94.39	91.25 93.21	90.93 93.8	90.96	0.846	497.2
	NS	87.62	88.65	88.13			
SVM	Health YR	93.32 94.31	87.91 92.34	90.53 93.31	90.5	0.824	108.7
	NS	90.51	84.58	87.44			
Model	Class	PA(%)	Evaluation dataset			Kappa	CT(s)
			UA(%)	F1(%)	OA(%)		
FFCDNN	Health YR	96.58 93.29	96.97 93.49	96.77 93.39	93.62	0.866	221.9
	NS	90.51	90.86	90.68			
BIT-DNN	Health YR	84.01 85.27	94.17 92.45	88.8 88.71	87.11	0.832	239.8
	NS	82.55	84.22	83.38			
AlexNet	Health YR	88.85 82.02	86.46 22.53	87.64 82.27	84.38	0.764	397.7
	NS	84.48	81.94	83.19			
SVM	Health YR	80.85 82.83	80.81 84.11	80.83 83.47	79.64	0.695	86.9
	NS	75.51	73.74	74.61			

#### 5.1.2 Experiment 2: model applications on the actual Sentinel-2 images under natural field conditions

The second experiment aims to further evaluate the robustness and transferability of the proposed model on the actual Sentinel-2 images under natural field conditions. For this purpose, the pretrained models in the last section are directly used in the pixel-wise classification of yellow rust and nitrogen deficiency on the actual Sentinel-2 time series in Ningqiang and Shunyi, and the ground truth samples are used as validation. The accuracy assessments of the pretrained SVM, CNN, and FFCDNN are listed in **Table 3**. In the comparison of the classification results in **Table 2**, it is clear that the proposed FFCDNN achieves the best and the most robust classification for the multiple plant stresses; the overall accuracy (i.e., 91.14% for Ningqiang and 91.63% for Shunyi) is consistent with the model evaluation results under controlled conditions (**Table 2**). In addition, the computing efficiency is highest among the deep learning-based baseline models. Overall, these results suggest that the proposed FFCDNN provides a more stable and robust performance than the baseline models for rapid noninvasive detection of plant stress in a fully automated and reproducible manner.

**Table 3:** The accuracy assessment of the pretrained models on actual Sentinel-2 time series in terms of producer’s accuracy (PA), user’s accuracy (UA), F1 score (F1), overall accuracy (OA), Kappa, and computing time (CT).

Model	Class	PA(%)	Ningqiang			Kappa	CT(s)
			UA(%)	F1(%)	OA(%)		
FFCDNN	Health YR	90.96 90.76	94.18 92.94	92.54 91.84	91.14	0.847	554.8
	NS	88.32	89.68	88.99			
BIT-DNN	Health YR	86.66 82.97	89.24 80.96	87.93 81.95	82.66	0.801	799.6
	NS	77.88	78.25	78.06			
AlexNet	Health YR	83.59 81.54	79.54 79.12	81.51 80.31	80.04	0.786	1194.4
	NS	80.38	76.05	78.16			
SVM	Health YR	65.32 71.46	59.93 70.79	62.51 71.12	62.62	0.689	217.4
	NS	56.2	52	54.02			
Model	Class	PA(%)	Shunyi			Kappa	CT(s)
			UA(%)	F1(%)	OA(%)		
FFCDNN	Health YR	95.88 89.44	92.34 85.99	94.08 87.68	91.63	0.855	483.8
	NS	94.28	91.86	93.05			
BIT-DNN	Health YR	86.19 84.37	88.82 82.12	87.49 83.23	84.37	0.817	679.6
	NS	80.73	83.96	82.31			
AlexNet	Health YR	81.93 81.6	83.06 80.88	82.49 81.24	81.47	0.759	1095.4
	NS	80.25	81.09	80.67			
SVM	Health YR	77.07 76.99	74.17 72.61	75.59 74.74	72.67	0.707	173.8
	NS	68.51	66.65	67.57			

**Figure 7:**
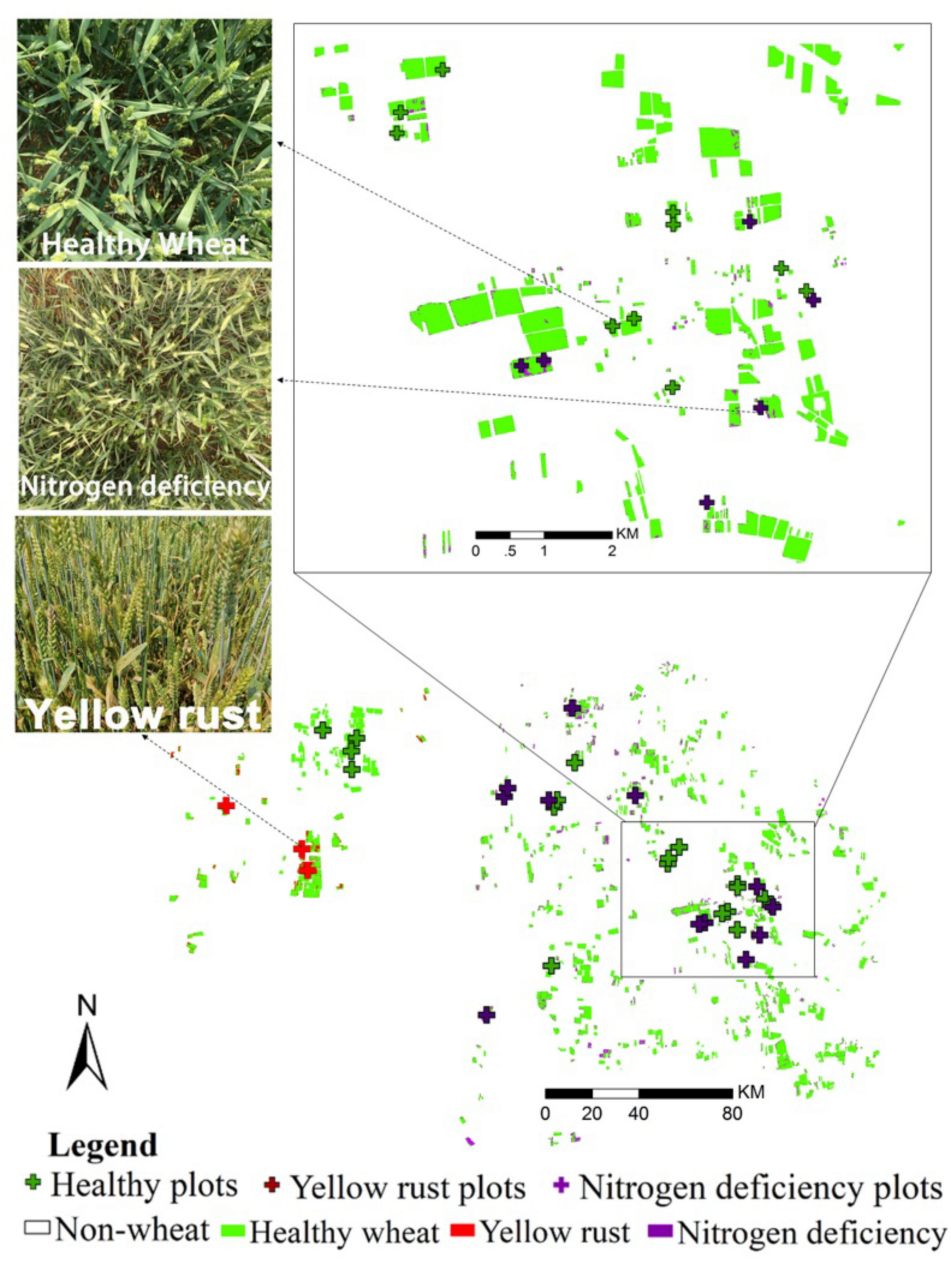
The occurrence monitoring and mapping of yellow rust in Ningqiang county, Shaanxi province (the zoomed in window shows the classification in the subregion).

**Figure 8:**
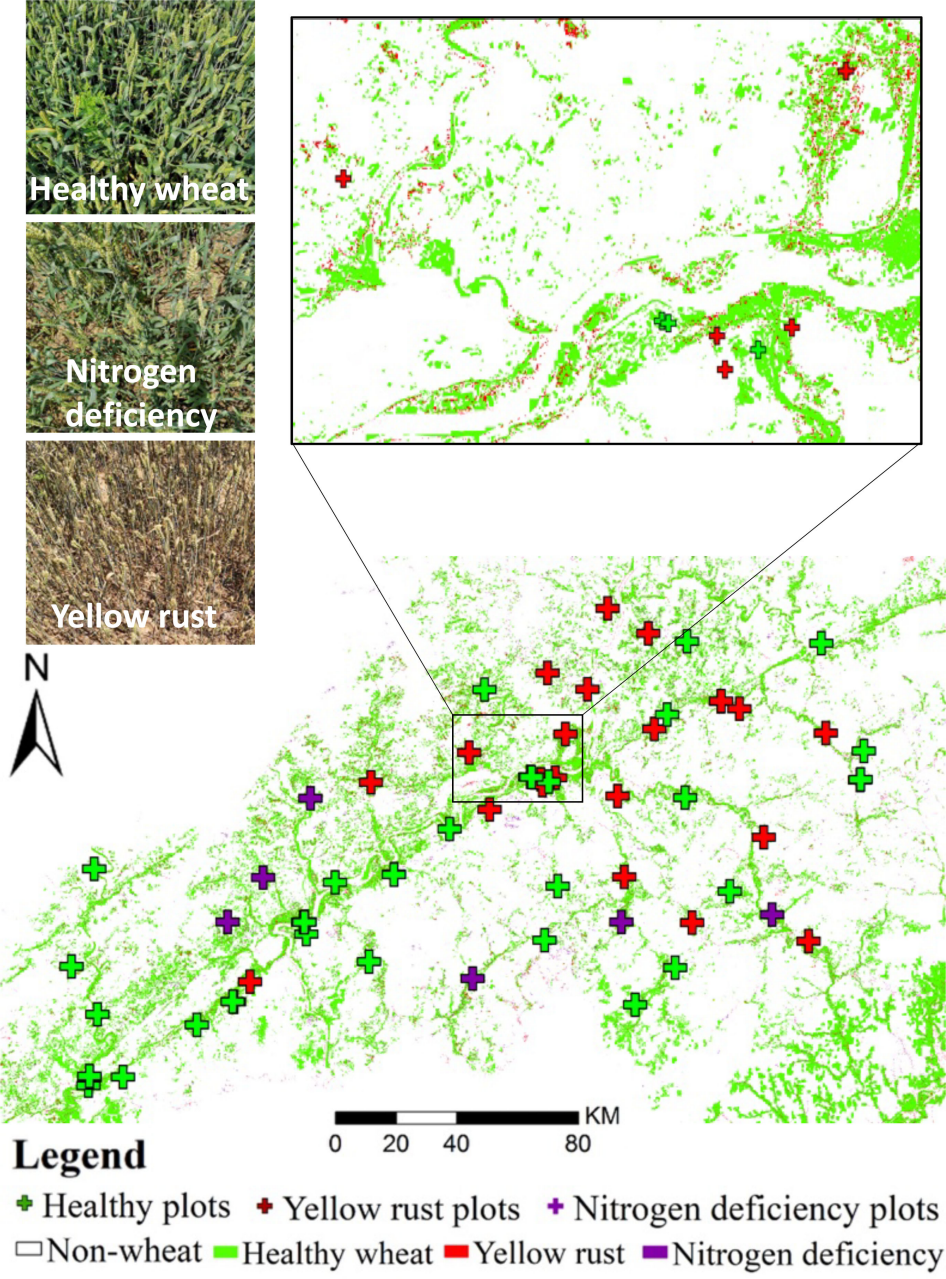
The detection and discrimination of yellow rust and nitrogen deficiency in Shunyi district, Beijing (the zoomed in window shows the classification in the subregion).

For the demonstration purpose, the FFCDNN-based classification maps of the yellow rust and nitrogen deficiency in Ningqiang and Shunyi are respectively illustrated in **Figures 7** and **8**. The spatial distributions of yellow rust and nitrogen deficiency in Ningqiang and Shunyi are consistent with our field survey. Specifically, for the Ningqiang case, yellow rust is mainly located around the river where ideal moisture is provided for the infestation and development of yellow rust (see the zoomed in window in **Figure 7**), and nitrogen deficiency is distributed around the edge of the county where the high transportation cast results in poor fertilization management. For the Shunyi case, nitrogen deficiency mainly occurs in the edge of the field patches (see the zoomed in window in **Figure 8**), and yellow rust slightly occurs in the west of the study area. These monitoring results are double-checked through telephone interviews with the local plant protection department.

### 5.2 The interpretability assessment of the model

Interpretability is one of the important matrices that measure bias and provide an explainable reason for prediction decisions from a model. In this study, the interpretability assessment mainly focuses on the data life in the proposed FFCDNN model and the representations of the intermediate features.

#### 5.2.1 The data life in the proposed FFCDNN model

**Figure 9:**
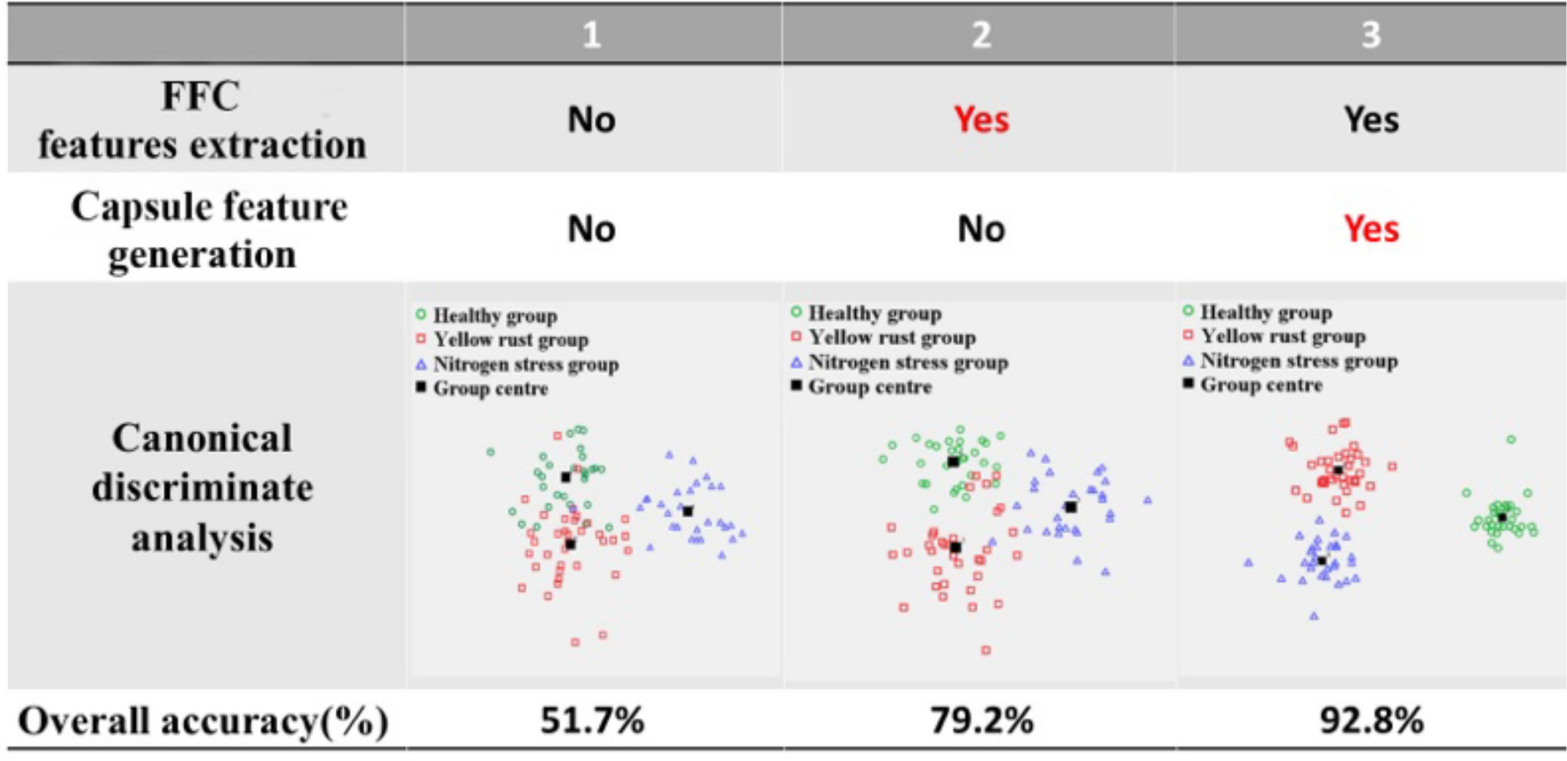
The visualization of the comparison for showing the effects of each module in FFCDNN on the canonical discriminate analysis and overall accuracy. Each column is a model with the modules on the top. Red highlights the main difference of the current model with the previous one.

In this study, two significant modules are proposed to characterize the yellow rust- and nitrogen deficiency-associated information from the Sentinel-2 time series, thus, 1) the FFC feature extraction and 2) the capsule feature generation. In order to evaluate the effects of each module on the inter-class separability, we conduct a canonical discriminate analysis to measure the clusters of the intermediate features. In the canonical discriminate analysis, the first two canonical discriminant functions are employed to establish the projective scatter plots. In addition, we gradually add the modules into the FFCDNN framework and compare their effects on classification accuracy. The visualization of the comparison is illustrated in **Figure 9**.

##### 5.2.1.1 The base model without the characterized modules

The base model architecture without the characterized modules is similar to a multilayer perception (MLP), thus, the *V I_LAI_* and *V I_LCC_* time series produced by the biological feature retrieval layer *L* ^(1)^ will directly input into the classifier *L* ^(5)^. The inter-class separability of the time series features is shown in the second column of **Figure 9**, and the overall accuracy achieved by the base model is approximately 51.7%.

##### 5.2.1.2 Adding the FFC layer

In the FFCDNN, the FFC feature extraction is the most important step to extract the yellow rust- and nitrogen deficiency-associated *V I_LAI_* and *V I_LCC_* frequency-domain features from the background noises. The canonical discriminate analysis indicates that by comparison with the time series features, the extracted frequency-domain features reveal the greater clusters between the different classes (the third column of **Figure 9**), and the overall accuracy reaches approximately 79.2%.

##### 5.2.1.3 Adding the capsule feature encoder

The capsule feature encoder is the most intelligent part of the proposed FFCDNN, which encapsulates the extracted scalar biological features into the vector features with the explicit biological representation of the target classes. The evident clusters and class edges can be figured out in the canonical projected scatter plot (the fourth column of **Figure 9**), and the final overall accuracy reaches 92.8%.

#### 5.2.2 The representations of the intermediate features

The primary contribution of this study is to model the part-to-whole relationship between the Sentinel-2-derived biological agents (i.e., *V I_LAI_* and *V I_LCC_*) and the specific stresses by encapsulating the scalar FFC features into the low-level class-associated vector structures. The philosophy behind the biologically composed features is that the vector features provide a hierarchical structure to represent the entanglement of the *V I_LAI_* and *V I_LCC_* fluctuations associated with yellow rust and nitrogen deficiency and provide evidence for the detection and discrimination of yellow rust and nitrogen deficiency.

**Figure 10:**
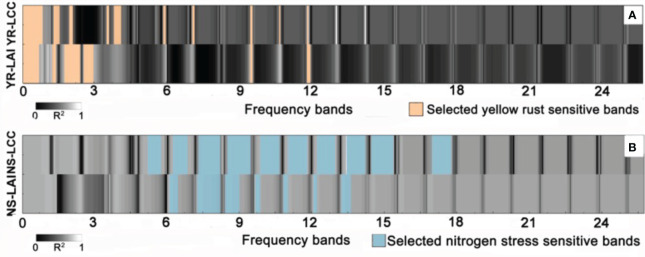
The coefficients of determination (*R* ^2^) between the components of the generated biologically composed features and the ground-measured severity of **(A)** yellow rust and **(B)** nitrogen deficiency.

The coefficients of determination (*R* ^2^) between the components of the generated biologically composed features and the ground-measured severity of yellow rust and nitrogen deficiency are calculated based on univariate correlation analysis (**Figure 10**). It is noted that according to Nyquist theorem, the maximum frequency component after FFT is 26 HZ; thus, the dimensionality of the generated biologically composed features will be less than 52. Our results illustrate that for yellow rust, both the *V I_LAI_* and *V I_LCC_* frequency features located in the low-frequency regions (2-4 HZ) highly relate with the severity levels of yellow rust, which means that the host–pathogen interaction of yellow rust may induce chronic impacts on the *V I_LAI_* and *V I_LCC_* fluctuation. These findings are in agreement with the biophysical and pathological characteristics of yellow rust that were reported in our previous study (Shi et al., 2018). For nitrogen deficiency, the associated *V I_LAI_* fluctuations are mainly located in the frequency regions of 5 15 Hz, and the associated *V I_LCC_* fluctuations are mainly located in the frequency regions of 6 13 Hz. This means that the nitrogen deficiency may give rise to more acute *V I_LAI_* and *V I_LCC_* responses than that of yellow rust on the Sentinel-2 time series. For instance, as reported in Behmann et al. (2014), the occurrence of nitrogen deficiency in green plants is associated with poor photosynthesis rates and further leads to abnormal LAI and LCC (i.e., reduced growth and chlorotic leaves). In conclusion, the proposed FFCDNN is able to capture periodic patterns and frequencies in the data directly during the learning process, making it more specialized for crop stress detection. In addition, by integrating FFTs into the model, FFCDNN can be more computationally efficient in scenarios where capturing frequency information is crucial for good performance.

## 6 Conclusion

The proposed FFCDNN model differs from existing approaches in the detection and discrimination of multiple plant stresses in the following three aspects: 1) Our model primarily considers plant biochemical information specific to the stresses. 2) The proposed FFC kernel represents the first attempt to use the FFT-based kernel in a deep neural network for biological dynamic extraction from the Sentinel-2 time series. 3) The well-designed capsule feature encoder demonstrates excellent performance in modeling the part-to-whole relationship between the extracted biological dynamics and the host–stress interaction. These three characteristics improve the interpretability of our model for decision-making, akin to human experts.

However, two challenges persist in the practical use of the proposed implementation. Firstly, the performance of our model is inherently limited by the accurate extraction of the biochemical prefilter. The Sentinel-2-based *V I_LAI_* and *V I_LCC_* estimations struggle to represent the real LAI and LCC values accurately, leading to the underestimation of the biological dynamics of specific stresses. Secondly, errors from the gap conditions and the co-registration of Sentinel-2 imagery introduce uncertainty in the modeling processes. These are the primary reasons for the performance decline in the practical application of the FFCDNN. Future research will investigate whether integrating information provided by multisource satellites into the FFCDNN framework could compensate for the LAI and LCC estimations and gap-related error, thereby further improving accuracies in detecting and discriminating yellow rust and nitrogen deficiency.

In conclusion, modeling the biochemical progress of specific plant stress is a key factor that influences the effectiveness of deep learning applications in the remote sensing detection and discrimination of multiple plant stresses. In this study, we proposed the FFCDNN model to analyze the stress-associated *V I_LAI_* and *V I_LCC_* biological responses from Sentinel-2 time series to achieve multiple plant classifications at the regional level. Comparisons with state-of-the-art models reveal that the proposed FFCDNN exhibits competitive performance in terms of classification accuracy, robustness, and generalization ability.

## Data availability statement

The original contributions presented in the study are included in the article/**Supplementary Material**. Further inquiries can be directed to the corresponding author/s.

## Author contributions

YS planned the study, designed the field experiments, developed the algorithm, and drafted the manuscript. LH and DD reviewed, edited, conducted interviews and supervised the manuscript and lead the revision. PG-M and WH prepared and conducted interviews, reviewed and edited the manuscript and conducted interviews. ZZ, YL and MH provided literature reviews, HM and MD reviewed and edited the manuscript. All authors improved the manuscript by responding to the review comments. All authors contributed to the article and approved the submitted version.
