# Supplementary material for: A fast Fourier convolutional deep neural network for accurate and explainable discrimination of wheat yellow rust and nitrogen deficiency from Sentinel-2 time series data
Source: Front Plant Sci. 2023 Oct 4;14:1250844. doi: 10.3389/fpls.2023.1250844 (PMC10582577; doi:10.3389/fpls.2023.1250844)
Supplement: Supplementary file 1 [file DataSheet_1.pdf]

Supplementary Material

1 SUPPLEMENTARY DATA

In this study, six popular narrow-band indices are selected as candidate proxies for LAI and LCC retrieval on Sentinel-2 bands. The detail information about these indices is listed in Table S1. Meanwhile, we also test the PROSAIL based look-up table (LUT) model, several parameters are set as the values that as same as **?**'s study in order to reduce the ill-posedness of the inversion problem.

The simulated Sentinel-2 reflectance under the controlled conditions are used to evaluate the performance of the selected vegetation indices on retrieval of LCC and LAI in their broad-band frame (Table **??**). All R2 values are significant at the 5% confidence level. In terms of LCC estimation, TCARI/OSAVI index reveals the optimal linear relationship in the entire observation period, with the average R^2 values of 0.695 and 0.688, the average RMSE of 3.13 and 2.82, for 2017 and 2018, respectively. In term of LAI estimation, WDVI reveals the strongest linear relationship, with the R^2 values on average of 0.807 and 0.823, the average RMSE of 0.2 and 0.19 for 2017 and 2018, respectively. The more visual contrast between measured and estimated LCC and LAI shown in Fig. S1 also reveals the similar results, thus the TCARI/OSAVI-derived VILCC and WDVI-derived VILAI have best correlations with the ground measured LCC and LAI (more close to the 1:1 line).

On the other hand, the LUT-based RTM inversion is a challenging exercise because a RTM has to be selected first, then a LUT has to be configured together with a cost function and finally regularization options have to be defined. These decisions may impact the inversion performance. Our results show that LCC is poorly retrieved with a R^2 value of 0.3369, the LAI retrieval is retrieved with a R^2 value of 0.5672 (Fig. S2). However, compared with the VIs-based estimation mentioned above, such inversion accuracies are still relatively lower. In addition, the LUT configuration is a supervised process, which will introduce noises into the model. In conclusion, we regard the TCARI/OSAVI-derived LCC and WDVI-derived LAI performed best in this study.

1.1 Figures

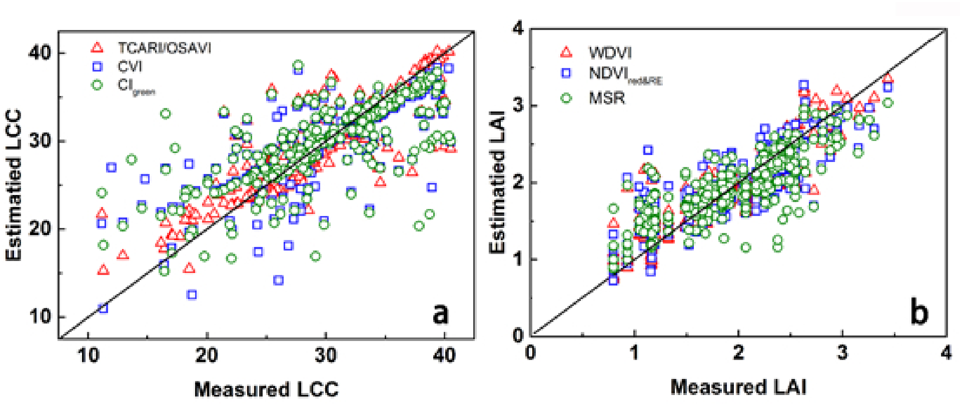

Figure S1. The relationship between measured and estimated (a) LCC and (b) LAI based on the pooled data of the simulated and actual Sentinel-2 bands under controlled field conditions.

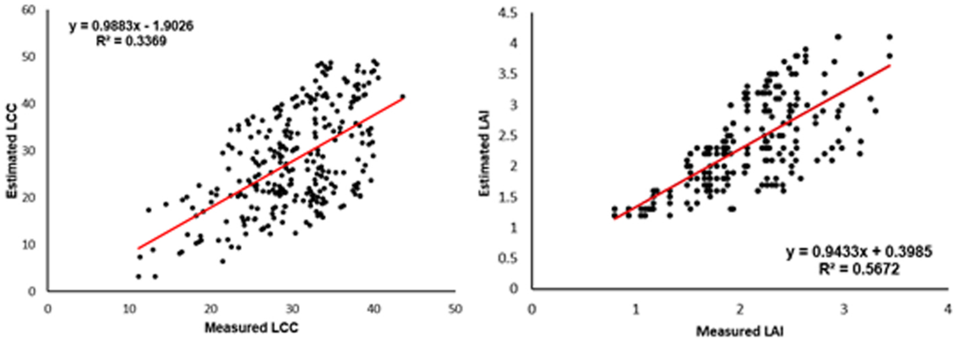

Figure S2. Measured vs. estimated LCC and LAI values based on the LUT-based RTM inversion scheme.

Table S1. Vegetation indices evaluated in this study to estimate leaf chlorophyll content and leaf area index

Crop parameter	Index	Formulation	Ref	Formulation for Sentinel-2 bands
LCC	TCARI/OSAVI	$\frac{3((R_{700}-R_{670})-0.2(R_{700}-R_{550})(R_{700}/R_{670}))}{1.16(R_{800}-R_{670})/(R_{800}+R_{670}+0.16)}$	?	$\frac{3((B5-B4)-0.2(B5-B3)(B5/B4)))}{1.16(B7-B4)/(B7+B4+0.16)}$
	CVI	$\frac{R_{870}/R_{550}}{R_{550}/R_{670}}$	?	$\frac{B8/B3}{B3/B4}$
	CI_{green}	$\frac{R_{780}}{R_{550}} - 1$	?	$\frac{B7}{B3} - 1$
LAI	$WDVI$	$R_{870} - C \times R_{670}$	?	$B8 - C \times B4$
	$NDVI_{red\&RE}$	$\frac{R_{870}-(a\times R_{670}+(1-a)\times R_{740})}{R_{870}+(a\times R_{670}+(1-a)\times R_{740})}$	?	$\frac{B8-(a\times B4+(1-a)\times R_{740})}{B8+(a\times B4+(1-a)\times B6)}$
	MSR	$\frac{R_{870}/R_{740}-1}{\sqrt{R_{870}/R_{740}+1}}$	?	$\frac{B8/B6-1}{\sqrt{B8/B6+1}}$

Table S2. Coefficient of determination (R^2) between the simulated Sentinel-2 bands-based vegetation indices and actual measured crop variables during the different day after treatment (DAT) under field controlled conditions.

	$TCARI/OSAVI - LCC$				$CVI - LCC$				$CI_{green} - LCC$			
	2017		2018		2017		2018		2017		2018	
DAT	R^2	RMSE	R^2	RMSE	R^2	RMSE	R^2	RMSE	R^2	RMSE	R^2	RMSE
7	0.51	3.3	0.52	2.63	0.41	2.67	0.4	2.92	0.38	3.85	0.41	2.71
14	0.67	3.35	0.69	2.14	0.5	2.04	0.49	2.73	0.46	2.77	0.48	3.2
21	0.66	2.91	0.62	1.87	0.65	2.17	0.62	2.89	0.58	2.55	0.61	2.66
28	0.73	2.29	0.7	2.35	0.76	2.59	0.77	2.5	0.69	2.81	0.68	2.31
32	0.75	2.29	0.72	2.12	0.77	2.19	0.79	3.1	0.69	2.98	0.69	3.15
35	0.77	1.28	0.76	1.88	0.75	2.7	0.79	2.47	0.68	2.92	0.64	3.2
42	0.77	1.64	0.76	2.2	0.68	2.67	0.71	2.57	0.6	3.05	0.65	2.77
49	-	-	0.72	2.45	-	-	0.68	2.57	-	-	0.64	2.9
Pooled	0.7	3.13	0.7	2.82	0.63	2.43	0.61	3.39	0.58	2.27	0.54	2.69
	$WDVI - LAI$				$NDVI_{red\&RE} - LAI$				$MSR - LAI$			
	2017		2018		2017		2018		2017		2018	
DAT	R^2	RMSE	R^2	RMSE	R^2	RMSE	R^2	RMSE	R^2	RMSE	R^2	RMSE
7	0.68	0.25	0.69	0.19	0.62	0.32	0.67	0.25	0.64	0.27	0.59	0.26
14	0.71	0.23	0.73	0.11	0.67	0.28	0.64	0.25	0.58	0.34	0.62	0.29
21	0.84	0.21	0.88	0.17	0.81	0.26	0.85	0.24	0.78	0.27	0.76	0.26
28	0.88	0.16	0.85	0.19	0.83	0.2	0.86	0.16	0.78	0.3	0.82	0.23
32	0.85	0.17	0.87	0.17	0.78	0.23	0.77	0.29	0.73	0.26	0.75	0.24
35	0.81	0.2	0.79	0.25	0.75	0.23	0.73	0.24	0.7	0.25	0.67	0.26
42	0.88	0.17	0.89	0.23	0.82	0.26	0.78	0.22	0.72	0.25	0.72	0.24
49	-	-	0.89	0.22	-	-	0.77	0.23	-	0.26	0.75	0.22
Pooled	0.8	0.21	0.82	0.22	0.74	0.24	0.72	0.25	0.65	0.24	0.64	0.35
